# SbbR/SbbA, an Important ArpA/AfsA-Like System, Regulates Milbemycin Production in *Streptomyces bingchenggensis*

**DOI:** 10.3389/fmicb.2018.01064

**Published:** 2018-05-23

**Authors:** Hairong He, Lan Ye, Chuang Li, Haiyan Wang, Xiaowei Guo, Xiangjing Wang, Yanyan Zhang, Wensheng Xiang

**Affiliations:** ^1^State Key Laboratory for Biology of Plant Diseases and Insect Pests, Institute of Plant Protection, Chinese Academy of Agricultural Sciences, Beijing, China; ^2^School of Life Sciences, Northeast Agricultural University, Harbin, China

**Keywords:** *Streptomyces bingchenggensis*, milbemycins, SbbR, SbbA, transcriptional regulation

## Abstract

Milbemycins, a group of 16-membered macrolide antibiotics, are used widely as insecticides and anthelmintics. Previously, a limited understanding of the transcriptional regulation of milbemycin biosynthesis has hampered efforts to enhance antibiotic production by engineering of regulatory genes. Here, a novel ArpA/AfsA-type system, SbbR/SbbA (SBI_08928/SBI_08929), has been identified to be involved in regulating milbemycin biosynthesis in the industrial strain *S. bingchenggensis* BC04. Inactivation of *sbbR* in BC04 resulted in markedly decreased production of milbemycin, while deletion of *sbbA* enhanced milbemycin production. Electrophoresis mobility shift assays (EMSAs) and DNase I footprinting studies showed that SbbR has a specific DNA-binding activity for the promoters of *milR* (the cluster-situated activator gene for milbemycin production) and the bidirectionally organized genes *sbbR* and *sbbA*. Transcriptional analysis suggested that SbbR directly activates the transcription of *milR*, while represses its own transcription and that of *sbbA*. Moreover, 11 novel targets of SbbR were additionally found, including seven regulatory genes located in secondary metabolite biosynthetic gene clusters (e.g., *sbi_08420*, *sbi_08432*, *sbi_09158*, *sbi_00827*, *sbi_01376*, *sbi_09325*, and *sig24_sbh_*) and four well-known global regulatory genes (e.g., *glnR_sbh_*, *wblA_sbh_*, *atrA_sbh_*, and *mtrA/B_sbh_*). These data suggest that SbbR is not only a direct activator of milbemycin production, but also a pleiotropic regulator that controls the expression of other cluster-situated regulatory genes and global regulatory genes. Overall, this study reveals the upper-layer regulatory system that controls milbemycin biosynthesis, which will not only expand our understanding of the complex regulation in milbemycin biosynthesis, but also provide a basis for an approach to improve milbemycin production via genetic manipulation of SbbR/SbbA system.

## Introduction

*Streptomyces* species are important sources for commercially available antibiotics (Kitani et al., 2011; Niu et al., 2016). The production of these antibiotics is typically specified by large gene clusters that usually include cluster-situated regulators (CSRs). In many cases, transcription of antibiotic biosynthetic gene clusters is under the direct regulation of CSRs, which are in turn controlled by a plethora of higher-level regulatory systems that respond to various environmental and physiological signals (Bibb, 2005; van Wezel and McDowall, 2011; Liu et al., 2013).

Milbemycins, a group of 16-membered macrolide antibiotics, were isolated from the culture broths of several *Streptomyces* species, including *Streptomyces hygroscopicus* subsp. *aureolacrimosus*, *Streptomyces thermoarchaensis*, and *Streptomyces bingchenggensis* (Rugg et al., 2005; Xiang et al., 2007). Similar to avermectins, milbemycins possess a lactone ring and are potent anthelmintic and insecticidal agents that are widely used in veterinary, agricultural, and medical fields (Shoop et al., 1995; Danaher et al., 2012; Jacobs and Scholtz, 2015). In particular, milbemycins have been used to control avermectin and organophosphorus pesticides-resistant mites, Liriomyza, aphidoidea, and aleyrodidae on more than two dozen plant species. Milbemectin, a mixture of milbemycin A3 and A4, shows high acaricidal activity and has been widely used against agricultural mites since 1990 (Pluschkell et al., 1999). The semisynthetic derivative of milbemycin A3/A4, milbemycin oxime, has been used to treat pests against filarid and nematode (Shoop et al., 1995; Danaher et al., 2012). Moreover, other milbemycin A3/A4 derivatives, such as lepimectin and latidectin, have also been commercialized and widely used in agricultural field (Ikari, 2013; Kim et al., 2016).

The species *S. bingchenggensis*, isolated in 2007 by our group, has been used for the industrial production of milbemycin (Gao et al., 2007; Wang et al., 2010). Given the importance of milbemycin A3/A4 and their derivatives, many efforts have been made to increase the titer of milbemycin. In *S. bingchenggensis*, a variety of mutagenic methods, including treatment with *N*-methyl-*N*′-nitro-*N*-nitrosoguanidine (NTG) (Wang et al., 2009), ultraviolet mutagenesis (Wang et al., 2009), as well as room temperature plasma (ARTP) mutations have been adopted to increase the titer of milbemycin A3/A4 (Wang et al., 2014). Moreover, *in silico*-based metabolic engineering strategies such as deletion of *milD* (encoding C5-O-methyltransferase) to remove undesired C5-O-methylmilbemycins and deletion of the nanchangmycin gene cluster, which putatively competes for the same precursors, such as malonyl-CoA and methylmalonyl-CoA, have been performed to enhance the production of milbemycin A3/A4 in *S. bingchenggensis* (Zhang J. et al., 2013). However, a limited understanding of the transcriptional regulation of milbemycin production has impeded the efforts to further improve antibiotic titers via manipulation of regulatory genes. Identification and characterization of novel transcriptional regulators involved in milbemycin biosynthesis are essential for the elucidation of the underlying regulatory mechanism, which in turn will benefit the genetic engineering of *S. bingchenggensis* to generate new hyper-producer strains.

In 2010, the milbemycin biosynthesis gene cluster (*mil*) was identified from the genome of *S. bingchenggensis* (Wang et al., 2010). The *mil* cluster (*sbi_00726*–*sbi_00790*) comprised of 10 genes, including one regulatory gene (*milR*) and nine structural genes located in six operons (i.e., *milA2*-*C*, *milA4*-*E*, *milR*-*A3*, *milA1*-*D*, *milF*, and *orf1*), and a large (62 kb) insertion fragment between *milR* and *milA1* (Wang et al., 2010; Zhang et al., 2016). *milR*, the only CSR gene in the *mil* cluster, encodes a large ATP-binding regulator of the LuxR (LAL) family. Previously, MilR was found to be essential for milbemycin production, and activates the expression of *milA4*-*E* and *milF* directly (Zhang et al., 2016). However, the higher-level transcriptional regulators controlling *milR* expression and, thus, the detailed regulatory networks of milbemycin production, remain elusive.

In *Streptomyces*, antibiotic production is often regulated by low-molecular-weight compounds, such as γ-butyrolactone signals (GBLs), avenolides, and furans (Bibb, 2005; O’Rourke et al., 2009; Kitani et al., 2011; Niu et al., 2016). GBLs, the largest group of these compounds, can elicit antibiotic biosynthesis at nanomolar concentrations by binding to cognate receptor proteins (Kitani et al., 2011). They share a characteristic 2,3-disubstituted-γ-butyrolactone core but differ in the C2 side chains that determine signaling specificity (Takano, 2006). In the process of cell growth, a small increase in expression of the GBL synthase, leads to gradually accumulated GBLs, which in turn binds to the receptor protein and releases its interaction with target genes (Mehra et al., 2008). To date, the well-studied GBL systems include A-factor-mediated ArpA/AfsA system in *Streptomyces griseus* (Khokhlov et al., 1967), SCBs-mediated ScbR/ScbA system in *Streptomyces coelicolor* (Cuthbertson and Nodwell, 2013), VBs-mediated BarA/BarX system in *Streptomyces virginiae* (Kinoshita et al., 1997), IM-2-mediated FarA/FarX system in *Streptomyces lavendulae* (Waki et al., 1997), and SVB1-mediated JadR3/JadW1 system in *Streptomyces venezuelae* (Zou et al., 2014). Given the large number of more than 900 *Streptomyces* species described and the very few GBL-mediated ArpA/AfsA-like systems known, further research on the ArpA/AfsA-like regulatory cascades is required (Niu et al., 2016).

In this report, we describe a putative ArpA/AfsA-like system, SBI_08928/SBI_08929, which is designated as SbbR/SbbA in *S. bingchenggensis.* SbbR is a homolog of GBL receptors, while SbbA resembles proteins involved in the production of GBL signals. We demonstrate that the SbbR/SbbA system plays an important role in the production of milbemycin. SbbR controls milbemycin production by directly activating the transcription of *milR*, while SbbA appears to have a repressive effect on milbemycin production; both SbbR and SbbA have positive effects on cell growth. Interestingly, SbbR is also a pleiotropic regulator that controls the transcription of other CSR genes and global regulatory genes. This work deepens our understanding of an upper-level of regulation in milbemycin biosynthesis, and provides an effective approach to improve milbemycin production via genetic manipulation of SbbR/SbbA system in *S. bingchenggensis*.

## Materials and Methods

### Bacterial Strains, Plasmids, Primers, and Growth Conditions

Bacterial strains and plasmids used in this study are summarized in **Table 1**; primers are listed in Supplementary Table S1. *S. bingchenggensis* BC04, the producer of milbemycin A3/A4, is derived from *S. bingchenggensis* CGMCC 1734 which is deposited at China General Microbiological Culture Collection (CGMCC). *S. bingchenggensis* BC04 and its derivatives were grown at 28°C on SKYM (0.4% sucrose, 0.1% skimmed milk powder, 0.2% yeast extract, and 0.5% malt extract pH 7.2) agar for spore collection. Besides, SKYM medium together with minimal medium (MM) (0.1% L-asparagine, 0.1% K_2_HPO_4_, 0.04% MgSO_4_⋅7H_2_O, 0.002% FeSO_4_⋅7H_2_O, 2% glucose, and 2% agar) was used to analyze morphological differentiation. The media and methods used for milbemycin production were the same as previously reported (Zhang et al., 2016).

**Table 1:** Strains and plasmids used in this study.

Strain or plasmid	Description	Source or reference
***S. bingchenggensis* strains**		
BC04	Parental strain; milbemycin producer	Laboratory stock
ΔsbbR	*sbbR* deletion mutant	This study
ΔsbbR/sbbR	ΔsbbR with the complementation vector pSET152::*sbbR*	This study
ΔsbbA	*sbbA* deletion mutant	This study
ΔsbbA/sbbA	ΔsbbA with the complementation vector pSET152::*sbbR*	This study
BC04/pSET152	Parental strain carrying empty vector pSET152	This study
***E. coli* strains**		
JM109	Host strain for cloning	Novagen
ET12567(pUZ8002)	ET12567 containing the non-transmissible RP4 derivative plasmid pUZ8002	Kieser et al., 2000
BL21 (DE3)	Host for protein expression	Novagen
DH5α	Host for GFP reporter system	Novagen
**Plasmids**		
pBluescript (KS+)	Routine DNA cloning and subcloning vector	Novagen
pUC119::*neo*	Obtaining kanamycin resistance gene (*neo*)	Zhang Y. et al., 2013
pKC1139	*E. coli*-*Streptomyces* shuttle vector	Bierman et al., 1992
pSET152	Integrative *E. coli*-*Streptomyces* shuttle vector	Kuhstoss et al., 1991
pET-23b (+)	Vector for His-tagged protein expression in *E. coli*	Novagen
pKC1139::Δ*sbbR::neo*	*sbbR* deletion vector based on pKC1139	This study
pSET152::*sbbR*	*sbbR* complemented vector based on pSET152	This study
pKC1139::Δ*sbbA::neo*	*sbbA* deletion vector based on pKC1139	This study
pSET152::*sbbA*	*sbbA* complemented vector based on pSET152	This study
pET23b::*sbbR*	*sbbR* expression vector based on pET23b	This study
pBmilR	A 413-bp DNA fragment containing SbbR binding region with the intact conserved site was inserted into pBluescript (KS+)	This study
pBsbbA	A 413-bp DNA fragment containing SbbR binding region with the intact conserved site was inserted into pBluescript (KS+)	This study
mupBmilR	A plasmid containing mutated conserved site amplified by PCR from pBmilR using primers mupmilR-F	This study
mupBsbbA	A plasmid containing mutated conserved site amplified by PCR from pBmilR using primers mupsbbA-F	This study
pSET152::P*_milR_gfp*	pSET152 derivative; containing P*_milR_*-driven *gfp*	This study
pSET152::P*_sbbA_gfp*	pSET152 derivative; containing P*_sbbA_*-driven *gfp*	This study
pSET152::P*_milR_gfp*::SF14*sbbR*	pSET152::P*_milR_gfp* derivative; containing PSF14-driven *sbbR*	This study
pSET152::P*_sbbA_gfp*::SF14*sbbR*	pSET152::P*_sbbA_gfp* derivative; containing PSF14-driven *sbbR*	This study


### Construction of the Phylogenetic Tree

The amino acid sequences of SbbR with its 36 homologs were individually aligned with multiple sequences obtained from the GenBank/EMBL/DDBJ databases using CLUSTAL X 1.83 software. The phylogenetic tree was reconstructed with the neighbor-joining algorithms (Saitou and Nei, 1987) using MEGA software version 6.06 (Tamura et al., 2013). Phylogenetic distances were calculated with the Jones-Taylor-Thornton (JTT) model (Wang et al., 2008). The stability of the clades in the trees was appraised using a bootstrap procedure with 1000 repeats (Felsenstein, 1985). All positions containing gaps and missing data were eliminated from the dataset (complete deletion option).

### Construction of *sbbR* and *sbbA* Disruption Mutants and Their Complementation

The *sbbR* and *sbbA* gene disruption were performed by the method of homologous recombination. To construct the *sbbR* disruption mutant, two fragments (sbbR-L: 1.9 kb, sbbR-R: 2.0 kb) flanking *sbbR* were amplified with the *S. bingchenggensis* BC04 genomic DNA as template by polymerase chain reaction (PCR) using the primer pairs sbbRLF/R and sbbRRF/R (Supplementary Table S1). The fragment sbbR-L was cut with *Hin*dIII and *Xba*I, and the fragment sbbR-R was cut with *Kpn*I and *Eco*RI. The plasmid pUC119::*neo* was used to obtain the kanamycin-resistance gene (*neo*) after *Hin*dIII and *Kpn*I digestion. The three resultant DNA segments were ligated into the sites of *Eco*RI and *Hin*dIII in pKC1139 to give pKC1139::*sbbR::neo*, in which a 519-bp fragment of *sbbR* was replaced by *neo*. pKC1139*::sbbR::neo* was subsequently introduced into *S. bingchenggensis* BC04 via conjugation (Kieser et al., 2000). The exconjugants were selected on MS agar containing apramycin (Ahn et al., 2012). After incubation on SKYM solid medium at 37°C for 9 days, double cross-over recombinant strains were obtained by selection for kanamycin-resistant (Kan^R^) and apramycin-sensitive (Apr^S^) clones. The obtained *sbbR* disrupted strain, designated as ΔsbbR, was confirmed by PCR using primer VsbbR-F and VsbbR-R followed by DNA sequencing.

Complementation was performed with pSET152 carrying *sbbR* expressed from its own promoter. The primers CsbbR-F and CsbbR-R were used to amplify the 1301-bp DNA fragment containing the *sbbR* ORF and its upstream region from genomic DNA of BC04 (Supplementary Table S1). The obtained DNA fragment was inserted into *Eco*RI and *Xba*I sites of pSET152 to create *sbbR* complementation vector pSET152::*sbbR*, which was introduced into ΔsbbR by intergenic conjugation to obtain the complemented strain ΔsbbR/sbbR (Kuhstoss et al., 1991).

To construct a *sbbA* deletion mutant, two fragments (sbbA-L: 1.5 kb and sbbA-R: 2.0 kb) flanking *sbbA* were amplified by primers sbbALF/R and sbbARF/R using the genomic DNA of BC04 as template, respectively. The fragment sbbA-L was digested with *Hin*dIII/*Xba*I, while the sbbA-R fragment was digested with *Kpn*I/*Eco*RI. These two fragments together with *neo* digested with *Hin*dIII/*Kpn*I were inserted into the site of *Eco*RI and *Hin*dIII in pKC1139 to generate pKC1139::*sbbA::neo*, in which a 385-bp fragment of *sbbA* was replaced by *neo*. The *sbbA* mutants were obtained with the same method as described previously, and were confirmed by PCR with VsbbA-F and VsbbA-R as primers, followed by DNA sequencing. The *sbbA* disruption mutant was designated as ΔsbbA.

For complementation of ΔsbbA, a 1643-bp fragment containing the intact *sbbA* ORF and its putative promoter region was amplified by PCR using primers CsbbA-F and CsbbA-R. The *Xba*I-*Eco*RV digested DNA fragment was ligated into pSET152 to give the *sbbA* complementation vector pSET152::*sbbA*, which was introduced into ΔsbbR by conjugation to obtain the complemented strain ΔsbbA/sbbA.

The plasmid pSET152 was introduced into BC04 to generate control strain BC04/pSET152.

### HPLC Analysis of Milbemycin A3/A4

Milbemycin A3/A4 was analyzed by the method previously reported (Zhang et al., 2016). HPLC was performed by Agilent 1100 HPLC (Zorbax, SB-C18 column, 4.6 mm × 250 mm, 5 μm) at a flow rate of 1.0 ml/min with gradient from 0 to 100% of solvent B in 15 min (Solvent A: CH_3_CN:H_2_O:CH_3_OH = 7:1:2, v/v/v; Solvent B: CH_3_OH) and detected at 242 nm.

### Overexpression and Purification of SbbR-His_6_ Protein

To prepare the SbbR-His_6_ protein, primers proSbbR-F and proSbbR-R were used to amplify the *sbbR* gene (the stop codon was excluded) with the BC04 genomic DNA. The amplified PCR products were digested with *Nde*I/*Xho*I and cloned between the *Nde*I/*Xho*I sites of pET23b (+) to generate the overexpression plasmid pET23b::*sbbR*, which was verified by nucleotide sequencing and then introduced into *E. coli* BL21 (DE3) for protein expression. IPTG (final concentration 0.1 mM) was used to induce SbbR-His_6_ expression. The nickel-nitrilotriacetic acid (Ni-NTA) agarose chromatography was used to purify the SbbR-His_6_ based on the protocol of the manufacturer (Novagen). The Amicon Ultra 0.5-ml centrifugal filters (Millipore membrane, 3 kDa cut-off size) (Millipore) were used to concentrate the protein based on the protocol provided by the manufacturer. Concentration of the total recovered protein was determined by BCA protein assay Kit (Novagen) with a standard of Bovine Serum Albumin (BSA). The purified protein was stored in 5% glycerol at -70°C.

### Electrophoretic Mobility Shift Assays (EMSAs)

Electrophoresis mobility shift assays were carried out as described previously (Wei et al., 2014; Li et al., 2016). In brief, two fragments [P*_milR_* (413-bp) and P*_R-A_* (464-bp)] corresponding to the upstream region of *milR* and the intergenic region of *sbbR* and *sbbA* were generated by PCR from the genomic DNA of *S. bingchenggensis* BC04 with primer pairs pmilR-F/R and psbbR-A-F/R, respectively. Primers phrdB-F and phrdB-R were used to amplify the control probe P*_hrdB_* (443-bp). The other 79 primer pairs used to amplify promoter region probes are listed in Supplementary Table S1. The DNA probe (10 ng) was incubated with various concentrations of SbbR-His_6_ in 20 μl reaction mixture containing 20 mM Tris base, 2 mM dithiothreitol (DTT), 5 mM MgCl_2_, 0.5 μg calf BSA, and 5% (v/v) glycerol, 500 ng poly[d(I-C)] was added when necessary. After incubation at 25°C for 25 min, DNA-protein complexes and free DNA were separated by electrophoresis on non-denaturing 4% (wt/vol) polyacrylamide gel at 4°C with 0.5× TBE as the running buffer. After electrophoresis, DNA in the gel was stained with SYBR Gold nucleic acid gel stain for 30 min in 1.0× TBE and photographed under ultraviolet transillumination using Quantity One.

### DNase I Footprinting

In order to determine the binding sites of SbbR on P*_milR_* and P*_R-A_*, DNase I footprinting assays were carried out as described previously (Li et al., 2016). DNA probes were prepared by PCR using 5′ FAM fluorescence-labeled primers listed in Supplementary Table S1. After being purified from agarose gel, labeled DNA probe (200 ng) and protein with different concentrations of SbbR-His_6_ were added to a final reaction volume of 50 μl, and incubated for 25 min at 25°C. DNase I (Promega) digestions were carried out for 75 s at 25°C and stopped with 20 mM EGTA. After ethanol extraction and precipitation, the purified samples were loaded in an Applied Biosystems 3730 DNA genetic analyzer together with the internal-lane size standard ROX-500 (Applied Biosystems). The dye primer-based sequencing kit (Thermo) was used to further precisely determine the sequences after aligning the capillary electrophoresis results of reactions. The results were then processed with GeneMarker v2.2.0 software.

### Site-Directed Mutagenesis of SbbR Binding Sequences

To assess the specificity of SbbR on its binding sequence, site-directed mutagenesis was performed. Two fragments P*_milR_* and P*_R-A_* were ligated into the *Eco*RV-digested plasmid pBluescript KS (+) to generate plasmids pBmilR and pBsbbA, respectively. A fragment containing mutated P*_milR_* was amplified by primer pair mupmilR-F/R using the plasmid pBmilR as template. Similarly, the fragment containing mutated P*_R-A_* was amplified by primer pair mupsbbA-F/R using pBsbbA as template. These two fragments were self-ligated, generating mutant plasmids mupBmilR and mupBsbbA, respectively. The mutated sequences at the binding sites were further verified by DNA sequencing. Then mupBmilR and mupBsbbA were digested with *Hin*dIII/*Bam*HI to generate mutant probes P*_milR_*^∗^ and P*_R-A_*^∗^, respectively. The binding activity of SbbR-His_6_ to these probes was subsequently determined by EMSAs.

### RNA Isolation and Quantitative Real-Time PCR

Transcription levels of genes were compared between *S. bingchenggensis* BC04 and ΔsbbR by quantitative real-time PCR (qRT-PCR) analysis. For this purpose, total RNAs were isolated from cultures of *S. bingchenggensis* BC04 grown at 28°C at various time points (1, 2, 3, 5, and 9 days). RNA extraction was performed as described previously (Zhang Y. et al., 2013). The RNA sample was treated with RQ1 RNase-free DNase I (Promega) to remove genomic DNA. UV spectroscopy and agarose gel electrophoresis were used to examine the RNA quality and quantity. Synthesis of cDNA and subsequent qRT-PCR were essentially the same as described previously (Zhang Y. et al., 2013).

### Determination of Transcriptional Start Points of *milR, sbbR*, and *sbbA*

The transcriptional start points (tsp) of *milR*, *sbbR* and *sbbA* were determined by using a 5′ RLM-RACE Kit (Thermo) according to the manufacturer’s instructions. Briefly, total RNAs were prepared from a 3 days culture of *S. bingchenggensis* BC04 (for *milR* and *sbbR*) and a 2 days culture of ΔsbbR (for *sbbA*). First, total RNAs (10 μg) were successively treated with Calf Intestinal Alkaline Phosphatase (CIAP) and tobacco acid pyrophosphatase (TAP) and ligated with the 5′ RLM-RACE adapter using T4 RNA ligase. Reverse transcription was preceded by using M-MLV Reverse Transcriptase with random decamers supplied. A first round of PCR was performed with the cDNAs derived from RT Reactions using 5′ RLM-RACE outer primer and another gene specific-primer listed in Supplementary Table S1. To obtain a single specific band, a second round of PCR was performed with the original PCR product (25-fold dilution) as template, by using 5′ RLM-RACE inner primer and another nested primer listed in Supplementary Table S1. The final PCR product was inserted into pBluescript (KS+) and sent for sequencing.

### GFP Reporter Assay in *E. coli*

For construction of the reporter plasmid, the original plasmid pSET152 was cut with *Bam*HI and *Xba*I, and the promoters P*_milR_* and P*_sbbA_* were amplified with two primer pairs pmilRGFP-F/R and psbbAGFP-F/R, respectively. The green fluorescence gene (*gfp*) was amplified with primers GFP-F and GFP-R from pTAC (Yin et al., 2015). Prior to PCR amplification, primers pmilRGFP-R, psbbAGFP-R, and GFP-F were phosphorylated with T4 polynucleotide kinase to facilitate subsequent ligation reactions. P*_milR_* and P*_sbbA_* were digested with *Bam*HI and the *gfp* coding region was digested with *Xba*I. The P*_milR_* and P*_sbbA_* promoters and the *gfp* coding region were ligated into pSET152 which was cut with *Bam*HI/*Xba*I in a three-piece ligation reaction to generate pSET152::P*_milR_gfp* and pSET152::P*_sbbA_gfp*, respectively. To evaluate the regulatory effects of SbbR on P*_milR_* and P*_sbbA_* promoters, the strong constitutive promoter SF14 and the coding region of *sbbR* were amplified from plasmid pGusTSF14 and genomic DNA of BC04 with primers pSF14-F/pSF14-R and psbbRGFP-F/psbbRGFP-R, respectively (Yin et al., 2015). The plasmids pSET152::P*_milR_gfp* and pSET152::P*_sbbA_gfp* were digested with *Nhe*I and assembled with the SF14 promoter and the *sbbR* coding region to obtain the corresponding reporter plasmids pSET152::P*_milR_gfp*::SF14*sbbR* and pSET152::P*_sbbA_gfp*::SF14*sbbR*, respectively, which include, both SF14 promoter controlled *sbbR* and a *gfp* controlled by P*_milR_* or P*_sbbA_*, respectively. *sbbR* and *gfp* were placed in opposite orientations. These four plasmids together with the empty vector pSET152, which was used as a control, were each transferred to DH5α to detect green fluorescence (excitation at 485 nm; emission at 535 nm, Synergy H4 Multi-Mode Reader). All fluorescence values were normalized to growth rates (OD_600_). The average and standard deviation of three experimental replicates are presented by each value and error bar, respectively.

### Preparation of GBL Crude Extracts

The procedure used for preparation of GBL crude extracts was as described previously with slight modification (Kitani et al., 2011; Zou et al., 2014). A total of 100 ml culture broth of BC04 or ΔsbbA grown in seed medium for 2 days was extracted with an equal volume of ethyl acetate. The organic phase was dried in a vacuum evaporator and re-dissolved in 1 ml DMSO.

### Determination of Cell Dry Weight

Two-milliliter cell cultures were collected by vacuum filtration and dried at 55°C to a constant weight.

### Statistical Analysis

All experiments mentioned above were carried out at least three times, independently. The mean values were presented as ± SD (standard deviation). The Student’s *t*-test was used to analyze the data. *P* < 0.05 is used as a standard criterion of statistical significance.

## Results

### Identification of the ArpA/AfsA System SbbR/SbbA in *S. bingchenggensis*

**FIGURE 1:**
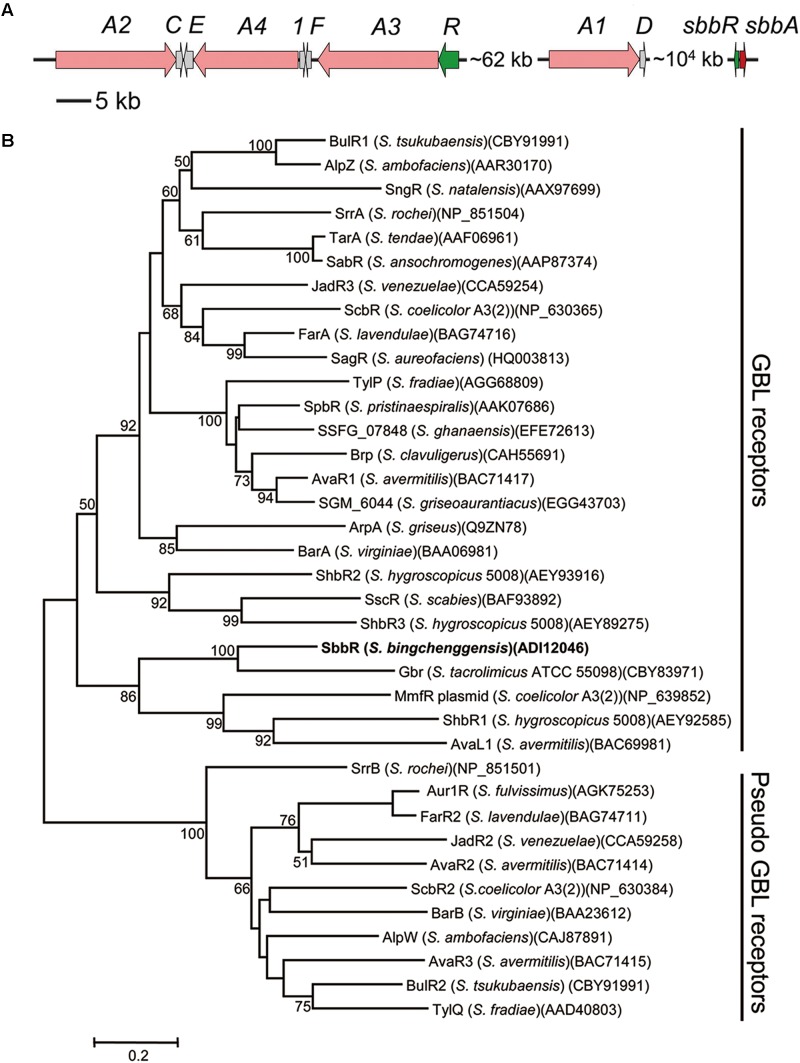
Bioinformatic analysis of SbbR in *S. bingchenggensis* BC04. **(A)** Schematic representation of the relative positions of *sbbR*, *sbbA*, and the *mil* cluster. Each arrow indicates a separate open reading frame (ORF) and orientation of transcription. **(B)** Phylogenetic analyses of SbbR and its homologs. SbbR of *S. bingchenggensis* BC04 is highlighted in bold. The CLUSTALW program was used for performing sequence alignment. The phylogenetic tree was constructed by applying the neighbor-joining algorithm. Bootstrap values >50% (based on 1000 replications) are shown at branch points. Bar, 0.02 substitutions per amino acid position for the phylogenetic tree of SbbR.

Genome analysis of *S. bingchenggensis* revealed an *arpA*/*afsA*-type system, *sbbR*/*sbbA* (*sbi_08928*/*sbi_08929*), which is ∼10^4^ kb far downstream of the *mil* cluster (*sbi_00726*–*sbi_00790*) (**Figure 1A**). An accurate prediction of open reading frame (ORF) is important for the study of gene function. After careful examination, we did not find a putative ribosome binding site (RBS) in the upstream region of the annotated *sbbR* or *sbbA* translation start codon; therefore, we re-annotated the coding sequences of *sbbR* and *sbbA*. The true *sbbR* coding region may span from nucleotide (nt) 10567682 to 10567062 on the reverse strand of the *S. bingchenggensis* chromosome consisting of 621 nucleotides, and *sbbA*’s real ORF may span from nt 10567842 to 10568729 on the forward strand of the chromosome consisting of 888 nucleotides.

*sbbR* encodes a TetR (Tetracycline Repressor) family regulator that shows similarity to GBL receptors such as Gbr from *Streptomyces tacrolimicus* (53% identity) (Salehi-Najafabadi et al., 2011), AvaR1 from *Streptomyces avermitilis* (31% identity) (Kitani et al., 2011), and JadR3 from *S. venezuelae* (30% identity) (Zou et al., 2014). *sbbA*, which is located upstream of the oppositely oriented *sbbR* gene, encodes a putative GBL biosynthesis protein that shows 32% identity with FarX from *S. lavendulae* (Waki et al., 1997), 31% identity with SrrX from *Streptomyces rochei* (Arakawa et al., 2007), 27% identity with AfsA from *S. griseus* (Khokhlov et al., 1967), and 25% identity with JadW1 from *S. venezuelae* (Zou et al., 2014). Previously, Nishida et al. (2007) constructed a phylogenetic tree for the GBL receptor homologs. These homologs were classified into two groups; one consists of canonical or “true” GBL receptors that bind GBLs as ligands, whereas the others are “pseudo” GBL receptors that do not bind GBLs. To get an indication of SbbR function, we reconstructed the phylogenetic tree for SbbR homologs. Phylogenetic analysis showed that SbbR belongs to the group of “true” GBL receptors represented by BarA (Kinoshita et al., 1997), ArpA (Khokhlov et al., 1967), and FarA (Waki et al., 1997) (**Figure 1B**). Furthermore, true GBL receptors and “pseudo” GBL receptors can be distinguished by their p*I* values: the true receptors appear to have acidic or neutral p*I* values, while the “pseudo” ones show basic p*I* values (Kitani et al., 2008). As SbbR has a p*I* value of 5.81, we hypothesize it belongs to the group of true GBL receptors, but this requires further experimental confirmation. In screening of the whole genome of *S. bingchenggensis*, no other homologs of SbbR or SbbA were discovered. This indicates that there is likely only one ArpA/AfsA-type system present in *S. bingchenggensis.*

### Effects of *sbbR* and *sbbA* Disruption on Phenotype and Milbemycin Production

To determine the roles of SbbR and SbbA in morphological differentiation and milbemycin production, *sbbR* and *sbbA* were disrupted individually. In ΔsbbR, a 519-bp fragment internal to *sbbR* ORF, containing the complete coding region of the HTH-type DNA-binding motif (a.a. 12–58) essential for TetR function, was replaced by a kanamycin resistance gene *neo* via homologous recombination (Supplementary Figure S1A). Similarly, the *sbbA* deletion mutant ΔsbbA was constructed with a 385-bp fragment covering much of the A-factor biosynthesis hotdog domain (a.a. 21–159) of *sbbA* replaced by *neo* (Supplementary Figure S1B). The resulting mutants ΔsbbR and ΔsbbA were confirmed by PCR (Supplementary Figures S1C,D) and DNA sequencing (data not shown).

**FIGURE 2:**
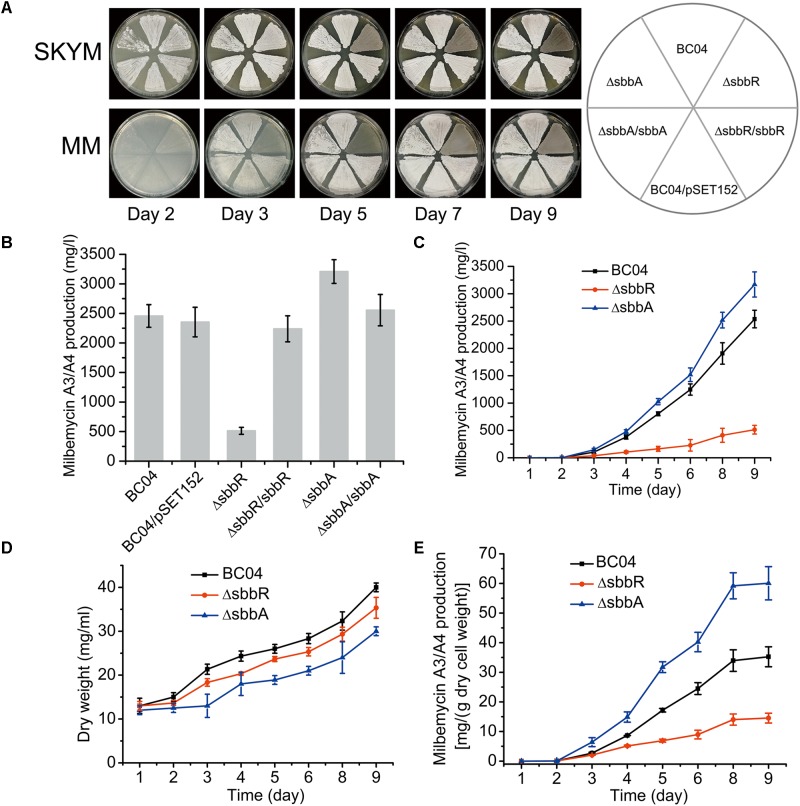
Effects of *sbbR* and *sbbA* on sporulation, milbemycin production, and cell growth. **(A)** Effects of *sbbR* and *sbbA* deletion on morphological differentiation. **(B)** Comparative milbemycin A3/A4 production in BC04, BC04/pSET152, ΔsbbR, ΔsbbR/sbbR, ΔsbbA, and ΔsbbA/sbbA cultured in fermentation medium. **(C)** Quantitative milbemycin A3/A4 production of BC04, ΔsbbR, and ΔsbbA cultured in fermentation medium. **(D)** Growth curves of BC04, ΔsbbR, and ΔsbbA strains cultured in fermentation medium. Biomass is expressed as dry cell weight. **(E)** Milbemycin A3/A4 yield per g dry cell weight of BC04, ΔsbbR, and ΔsbbA strains. Data are presented as the averages of the results of three independent experiments. Error bars show standard deviations.

Compared with the parental strain *S. bingchenggensis* BC04 and BC04/pSET152 controls, ΔsbbR formed spores earlier and the spores were darker pigmented on SKYM (a rich medium for sporulation) and MM; while on MM, ΔsbbR presented an obvious weak growth of aerial mycelium (**Figure 2A**). ΔsbbA grew a little sparse but sporulated normally on these two media (**Figure 2A**). In complementation experiments, pSET152::*sbbR* was introduced into ΔsbbR to generate ΔsbbR/sbbR and pSET152::*sbbA* was transferred to ΔsbbA to generate ΔsbbA/sbbA. As expected, the complementation strains gave full complementation (**Figure 2A**). These results suggested that *sbbR* and *sbbA* are involved in the regulation of morphological development.

In comparison with the parental strain *S. bingchenggensis* BC04, production of milbemycin A3/A4 in ΔsbbR was significantly reduced (**Figure 2B** and Supplementary Figure S2); whereas the titer of milbemycin A3/A4 in ΔsbbA was increased (**Figure 2B** and Supplementary Figure S2). To verify that the opposite phenotypic changes were due to the separate disruption of *sbbR* and *sbbA*, complementation strains ΔsbbR/sbbR and ΔsbbA/sbbA were also tested for milbemycin production. The results showed that ΔsbbR/sbbR and ΔsbbA/sbbA produced similar levels of milbemycin A3/A4 as those of BC04 and BC04/pSET152 controls, indicating that SbbR is an activator of milbemycin production and SbbA has a repressive effect on milbemycin production (**Figure 2B** and Supplementary Figure S2). Next, milbemycin production (**Figure 2C**), the cell growth (**Figure 2D**), and milbemycin yield per gram dry cell weight (**Figure 2E**) of ΔsbbR, ΔsbbA and the parental strain BC04 were quantitatively compared in fermentation medium. Compared with the parental strain BC04, deletion of *sbbR* decreased milbemycin production dramatically by 80% (from 2537 to 514 mg/l) and reduced cell growth; the disruption of *sbbA* increased milbemycin production by 25% (from 2537 to 3169 mg/l) but reduced biomass accumulation throughout the entire time-course (**Figures 2C,D**). These data revealed that *sbbR* plays a positive regulatory role in milbemycin production and cell growth; meanwhile, *sbbA* plays a negative role in milbemycin production and is also important for cell growth.

### SbbR Binds to the Upstream Regions of *milR, sbbR*, and *sbbA*

**FIGURE 3:**
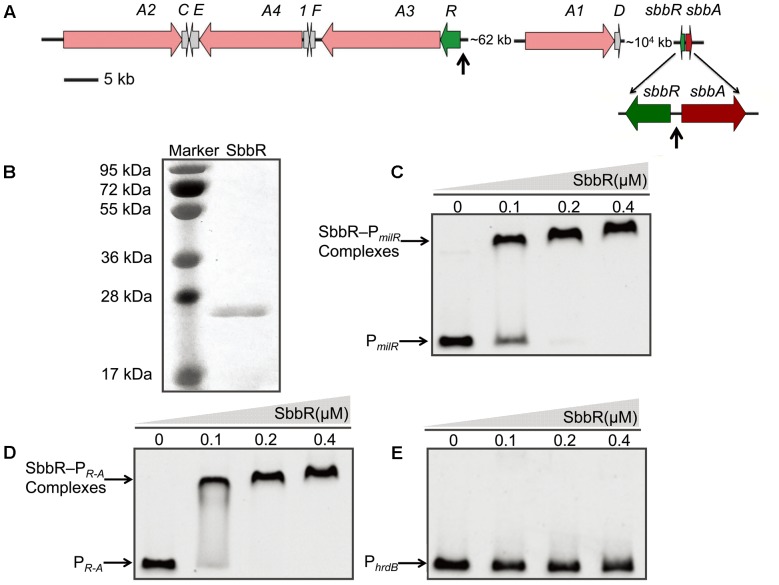
Binding of purified SbbR to the promoter regions of *milR*, *sbbR*, and *sbbA.* **(A)** Schematic representation of the relative positions of *sbbR*, *sbbA*, and the *mil* cluster. The vertical arrows indicate the binding region of SbbR. **(B)** SDS-PAGE analysis of the purified SbbR-His_6_ (23.6 kDa). **(C–E)** EMSAs of SbbR binding to the promoters P*_milR_*, P*_R-A_*, and P*_hrdB_*. P*_milR_*: the promoter region of *milR*. P*_R-A_*: the intergenic region of *sbbR* and *sbbA*. P*_hrdB_*: the promoter region of *hrdB*. Each lane contains 10 ng of DNA probes. The promoter regions of *milR*, the bidirectional *sbbR*/*sbbA* and *hrdB* were prepared as 413, 464, and 443 bp probes, respectively. DNA-protein complexes and free probes are indicated by arrows.

To assess whether the SbbR/SbbA system directly regulates milbemycin biosynthesis, the ArpA-like protein SbbR was expressed as a C-terminally His_6_-tagged protein and tested in EMSAs with all potential promoter regions in the *mil* cluster, including the putative promoters of *milA2*, *milA4*, *milE*, *orf1*, *milF*, *milR*, *milA3*, and *milA1* (**Figure 3A** and Supplementary Table S2). The intergenic region of *sbbR*-*sbbA* (P*_R-A_*) was also included for EMSA, because *sbbR* and *sbbA* are adjacent on the chromosome, transcribed divergently, and regulation of a neighboring oppositely arranged gene is a common feature of TetR-family regulators (Ahn et al., 2012; Yuan et al., 2016). Results from EMSAs showed that SbbR only formed complexes with P*_milR_* and P*_R-A_* in a concentration-dependent manner (**Figures 3C,D**), while 500-fold excess of non-specific poly (dI-dC) could not dissociate SbbR from P*_milR_* (DNA region upstream of *milR*) or P*_R-A_* (Supplementary Figure S3). Negative control P*_hrdB_* also showed no band-shift when SbbR concentration reaches 0.4 μM (**Figure 3E**). These results suggested that SbbR binds specifically to P*_milR_* and P*_R-A_*.

**FIGURE 4:**
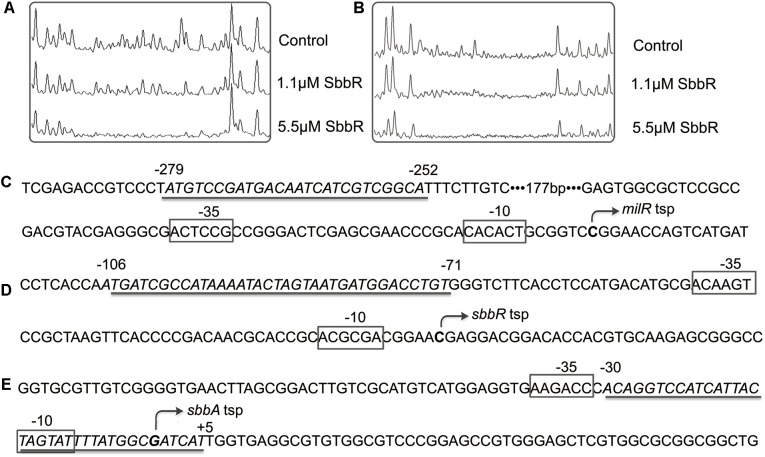
Binding sequences of SbbR on the promoter regions of *milR*, *sbbR*, and *sbbA*. **(A)** DNase I footprinting assay of SbbR binding site on *milR* promoter region (P*_milR_*). **(B)** DNase I footprinting assay of SbbR binding site on *sbbR* (*sbbA*) promoter region P*_R-A_*. Upper fluorograms: control reaction with 10 μM BSA. Protection fluorograms were obtained with increasing amounts of SbbR-His_6_. **(C–E)** Nucleotide sequences of the promoter regions of *milR*, *sbbR*, and *sbbA.* The tsp is marked by a bent arrow. The SbbR-binding sequences are underlined, and the putative –10 and –35 regions are marked by boxes. Numbers indicate distance (nt) from respective tsp.

To further define the accurate binding sequences of SbbR in the upstream region of *milR* and the intergenic region of *sbbR*-*sbbA*, the transcription start points (tsps) of *milR*, *sbbR*, and *sbbA* were determined by 5′ RACE. For *milR*, the tsp was identified at a C, 39 nt upstream of the *milR* translation start codon (tsc) (Supplementary Figure S4A). For *sbbR*, the tsp was identified at a C, 15 nt upstream of the *sbbR* tsc (Supplementary Figure S4B). For *sbbA*, the tsp was identified at a G, 43 nt upstream of the *sbbA* tsc (Supplementary Figure S4C). DNase I footprinting experiments with 5′ FAM labeled probes were carried out to define the protected sites of SbbR on its target promoters. As shown in **Figure 4**, SbbR protected a region spanning from nt -279 to -252 relative to the *milR* tsp (**Figures 4A,C**), far away from the -35 and -10 elements of *milR* promoter. The footprint of the *sbbR* promoter region covered a region from nt -106 to -71 (**Figures 4B,D**), and this region corresponds to positions -30 to +5 relative to the tsp of *sbbA* (**Figures 4B,E**). Analysis of these two protected sequences interacting with SbbR showed no consensus motif. However, after careful examination, a 6 nt imperfect inverted repeat (CCGAYG [Y=C or T]) with 8 bp spacing within the binding region of SbbR on P*_milR_*, and another different 6 nt inverted repeat (CCATHA [H=C or A]) with 11 bp spacing within the protected sequences of SbbR on P*_R-A_* were defined, respectively.

**FIGURE 5:**
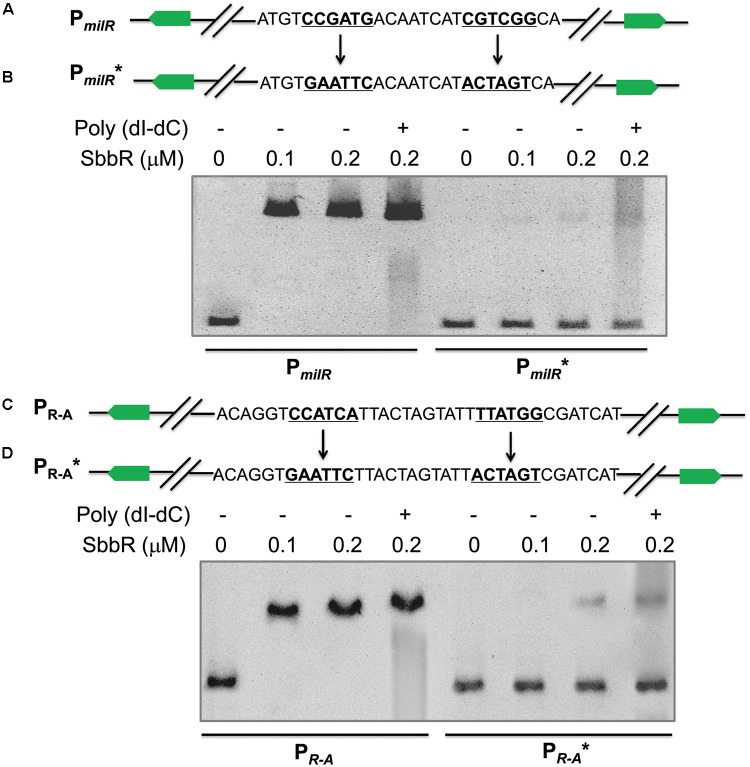
Mutational analysis of SbbR binding sites on P*_milR_* and P*_R-A_*. **(A)** Mutations introduced into the 6 nt inverted repeat of P*_milR_*. The changed nucleotides are underlined. **(B)** EMSAs of the wild-type probe P*_milR_* and mutated probe P*_milR_*^∗^. Each lane contains 10 ng of DNA probes. The concentrations of SbbR used in lanes 1 to 4 were 0, 0.1, 0.2, and 0.2 μM, respectively. Line 4 contains 500-fold poly(dI-dC). **(C)** Mutations introduced into the 6 nt inverted repeat of P*_R-A_*. The changed nucleotides are underlined. **(D)** EMSAs of the wild-type probe P*_R-A_* and mutated probe P*_R-A_*^∗^.

To determine the actual contributions of these two different inverted repeats to the binding activity of SbbR. P*_milR_* and P*_R-A_* were separately mutated to P*_milR_*^∗^ and P*_R-A_*^∗^. To obtain P*_milR_*^∗^, the 6 nt imperfect inverted repeat of P*_milR_* was replaced by *Eco*RI and *Spe*I restriction sites (**Figure 5A**); similarly, the 6 nt imperfect inverted repeat of P*_R-A_* was also replaced by *Eco*RI and *Spe*I restriction sites, generating P*_R-A_*^∗^ (**Figure 5C**). P*_milR_*^∗^ and P*_R-A_*^∗^ were then tested in EMSAs with SbbR. As shown in **Figure 5**, compared with the wild-type DNA probes, only a small amounts of complexes were formed for P*_milR_*^∗^ or P*_R-A_*^∗^ (**Figures 5B,D**). Thus, these two inverted repeats were critical in the binding of SbbR.

### *SbbR* Activates the Transcription of *milR* but Represses *sbbR* and *sbbA*

**FIGURE 6:**
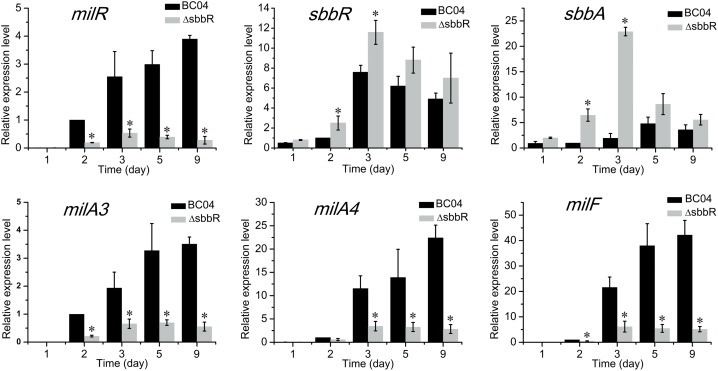
Quantitative real-time RT-PCR analysis of *milR*, *sbbR*, *sbbA*, *milA3*, *milA4*, and *milF* in BC04 and ΔsbbR. All RNA samples were isolated from 1, 2, 3, 5, and 9 days cultures. The transcriptional levels of related genes are presented relative to that of BC04 collected after fermentation for 24 h, which was arbitrarily assigned a value of 1. Data are presented as the averages of three independent experiments conducted in triplicate. 16S rRNA transcription was monitored and used as the internal control. Error bars show standard deviations. *P-*values were determined by Student’s *t-*test. ^∗^*P* < 0.05.

To examine the effect of SbbR on the transcription of its targets, RNA samples were extracted from the mycelia of BC04 and ΔsbbR cultivated for various days (1, 2, 3, 5, and 9 days). The transcription levels of *milR*, *sbbR* (the ORF of *sbbR* in ΔsbbR was partially left at its 5′ end) and *sbbA* were then assessed by quantitative real-time RT-PCR (qRT-PCR). The results showed that transcription of *milR* decreased significantly in ΔsbbR at all tested time points, whereas the transcript levels of *sbbR* and *sbbA* were shown to be higher in ΔsbbR than in BC04 (**Figure 6**). The transcription profiles of *sbbR* and *sbbA* were similar, reached the highest level at about 3 days of cultivation when milbemycin production was initiated, and then the transcripts decreased gradually (**Figure 6**). Since *milA3* is located in the same operon with *milR* (the *milR-A3* operon), and MilR activates the expression of the *milA4-E* operon and *milF* directly (Zhang et al., 2016); thus, transcription of *milA3*, *milA4*, and *milF* was also measured. Transcripts of these three genes were also decreased dramatically (**Figure 6**). These results suggested that SbbR regulates milbemycin biosynthesis by activating the transcription of *milR* directly, and SbbR is a direct repressor of its own transcription and that of *sbbA*.

**FIGURE 7:**
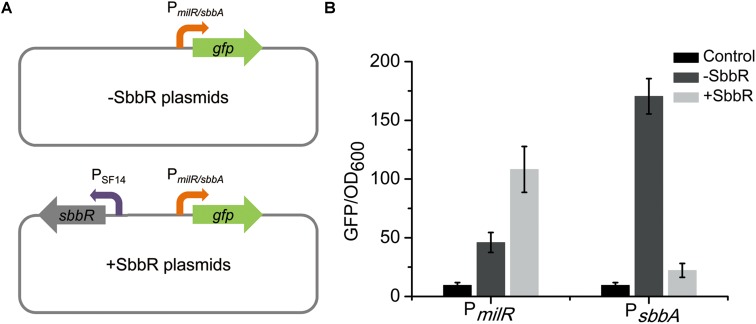
Analyses of regulatory relationships between SbbR and its target genes (*milR* and *sbbA*) in *E. coli*. **(A)** An illustration of the reporter plasmids. **(B)** Determination of the regulatory relationships between SbbR and its target promoters (P*_milR_* and P*_sbbA_*). All values are in relative fluorescence unit (GFP/OD_600_) and represent the averages of at least three independent readings.

The regulatory relationships between SbbR and its target genes (*milR* and *sbbA*) were further verified using a heterologous promoter-reporter system in *Escherichia coli* (Yin et al., 2015). As shown in **Figure 7A**, the promoters of *milR* and *sbbA* were cloned separately upstream of *gfp* [encoding green fluorescent protein (GFP)] to generate pSET152::P*_milR_gfp* and pSET152::P*_sbbA_gfp*, respectively. The resulting plasmids were then each transferred to *E. coli*. Afterwards, *sbbR* controlled by SF14 was assembled into the above two recombinant vectors to generate pSET152::P*_milR_gfp*::SF14*sbbR* and pSET152::P*_sbbA_gfp*::SF14*sbbR*, respectively, which were also introduced into *E. coli*. Then the four strains constructed above together with the control strain harboring pSET152 were detected for green fluorescence. Fluorescence in the strain harboring pSET152::P*_milR_gfp*::SF14*sbbR* was enhanced 2.4-fold compared to that in the strain harboring pSET152::P*_milR_gfp*, while fluorescence in pSET152::P*_sbbA_gfp*::SF14*sbbR*-containing strain exhibited a decrease of 87% compared to that of pSET152::P*_sbbA_gfp* strain (**Figure 7B**). These results agreed with the data obtained from transcriptional profiles in *S. bingchenggensis* BC04, and provided convincing evidence that SbbR activates the expression of *milR* and represses the transcription of *sbbA* directly.

### Identification of New *SbbR* Targets That Encode Transcriptional Regulatory Proteins

**FIGURE 8:**
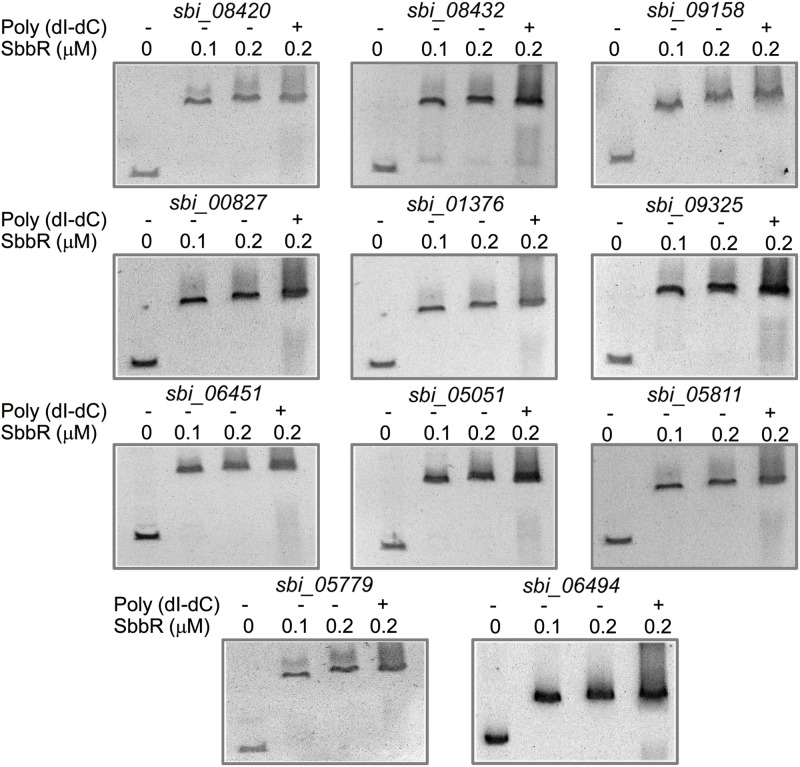
Electrophoresis mobility shift assays (EMSAs) of SbbR binding to the upstream regions of 11 novel targets. The amounts of SbbR (μM) used were as indicated and 10 ng of DNA probes was added to each EMSA reaction. Lane 2–4 contains 0.1, 0.2, and 0.2 μM SbbR and Line 4 contains 500-fold non-specific poly (dI-dC).

When analyzing the function of SbbR/SbbA system, it is possible that SbbR/SbbA may be involved in a more comprehensive regulation of diverse cellular processes directly or via regulatory cascades. As shown in **Figures 3, 4**, the promoter of *milR* and the intergenic region of *sbbR* and *sbbA* were the primary targets of SbbR; however, no highly conserved recognition sequence could be extracted from the two binding sites. The relatively unconserved binding sites for SbbR make it difficult to predict novel targets through *in silico* screening of the *S. bingchenggensis* genome sequence using MAST/MEME tool. Therefore, to further explore the SbbR/SbbA regulon, we selectively searched for binding targets of SbbR that encode transcriptional regulators. A total of 43 genes located in putative secondary metabolism clusters and 29 genes that encodes homologs of global or pleiotropic regulators were selected for EMSAs (Supplementary Table S2). The probes containing the upstream regions of the 72 genes were amplified (Supplementary Table S1). Subsequent EMSAs showed that SbbR has strong affinity for 11 DNA probes (**Figure 8**), and the deduced products of the putative target genes were as follows: two SARP family CSRs (SBI_08420 and SBI_08432) of nanchangmycin biosynthetic gene cluster (Wang et al., 2013), one SARP family CSR (SBI_09158) of actagardine biosynthetic gene cluster (Wang et al., 2013), one LAL family CSR (SBI_00827) of a cluster encoding non-ribosomal peptide synthases (Wang et al., 2013), one LAL family CSR (SBI_01376) of lasalocid biosynthetic gene cluster (Wang et al., 2013), one putative LAL family regulator (SBI_09325) of melanin biosynthetic gene cluster (Wang et al., 2013), one putative Sig24 homologous regulator (SBI_06451, Sig24_sbh_) of desferrioxamine biosynthetic gene cluster (Wang et al., 2013), one GlnR homolog (SBI_05051, GlnR_sbh_), one WblA-like protein (SBI_05811, WblA_sbh_), one AtrA homolog protein (SBI_05779, AtrA_sbh_), and one MtrA/B-type two-component system (TCS: SBI_06494/06495, MtrA/B_sbh_).

**FIGURE 9:**
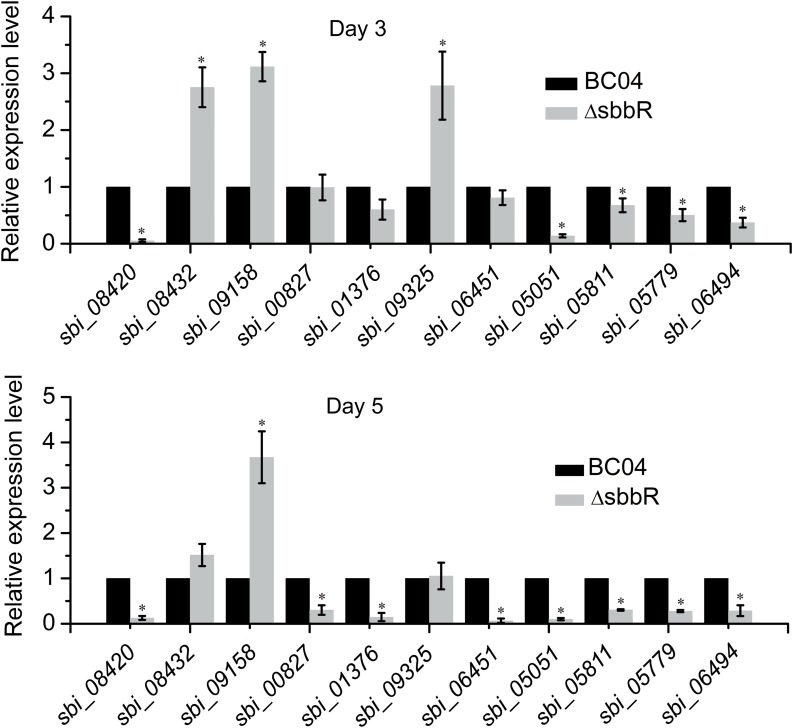
Quantitative real-time PCR (qRT-PCR) analysis examining the effects of *sbbR* deletion on the transcription of new SbbR target genes. RNA samples were isolated from 3 and 5 days cultures. The transcription of genes in BC04 was assigned a value of 1. 16S rRNA transcription was monitored and used as the internal control. Data are presented as the averages of three independent experiments conducted in triplicate. Error bars show standard deviations. ^∗^*P* < 0.05.

To investigate the regulatory role of SbbR in the transcription of these new target genes, qRT-PCR analysis was carried out to assess the transcription of related genes. As shown in **Figure 9**, SbbR repressed the transcription of two SARP-family regulatory genes (*sbi_08432* and *sbi_09158*) and one LAL-family regulatory gene *sbi_09325*, as the transcriptional levels of these genes were increased in ΔsbbR compared with BC04 at day 3 or both days (days 3 and 5). In contrast, transcription of the other eight genes was lower in ΔsbbR than in BC04 at day 5 or both days, indicating that SbbR is an activator of these genes (**Figure 9**). These data suggested that SbbR plays a differential regulatory role toward different target genes. It should be noted that these 11 novel targets of SbbR are identified only from the above 72 transcriptional regulatory genes that act as putative CSRs or pleiotropic regulators. Genome-wide identification of SbbR targets via chromatin immunoprecipitation followed by sequencing (Chip-seq) will be carried out in our future works.

### Effect of Culture Extracts From *S. bingchenggensis* on the Binding Activity of SbbR

In *S. bingchenggensis*, SbbR and SbbA constitute the quorum-sensing system, in which SbbR can respond to the likely GBL-type signaling molecules generated by SbbA. Thus, to determine whether SbbR is able to respond to small signaling molecules produced by *S. bingchenggensis*, ethyl acetate extracts of strains BC04 and ΔsbbA were prepared and assayed for their effect on the binding activity of SbbR to P*_milR_*. As expected, extracts from BC04 could dissociate SbbR from SbbR-P*_milR_* complexes, leading to about forty thousand inhibitory units/l (1 inhibitory units is defined as the activity of dissociating about half of 10 ng of P*_milR_* from SbbR) (Supplementary Figure S5). Unexpectedly, when added at a high concentration, extracts from ΔsbbA could also dissociate SbbR from SbbR-P*_milR_* complexes (Supplementary Figure S5). This indicated that SbbR could bind the factor determined by the GBL biosynthesis protein SbbA; it could also bind to some unknown ligands produced in ΔsbbA. It is interesting to probe the putative chemicals that bind to SbbR produced by ΔsbbA, which will be the focus of our future work. These results suggested that SbbR may be a genuine signaling molecular receptor protein, and it is possible that the molecules produced by SbbA regulate the biosynthesis of milbemycin via interactions with SbbR, but the further structural analysis is impeded by the fact that they are normally produced in very small quantities (Niu et al., 2016).

## Discussion

In the Gram-positive *Streptomyces*, ArpA/AfsA systems are important for antibiotic production and morphological differentiation (Tan et al., 2013; Zou et al., 2014). In the present study, genome analysis of sequenced *S. bingchenggensis* revealed a potential ArpA/AfsA-type system annotated as SbbR/SbbA. *sbbR* and *sbbA* are adjacent and divergently transcribed, but they locate far from the *mil* cluster. We demonstrate that SbbR, a GBL receptor homolog, activates the biosynthesis of milbemycin by directly activating the transcription of *milR*, the CSR of the *mil* cluster; while the GBL synthase homolog, SbbA, has a repressive effect on milbemycin production. Interestingly, both SbbR and SbbA have positive effects on cell growth, and SbbR also acts pleiotropically by controlling the expression of many other CSR genes and global regulatory genes.

Generally, it is thought that GBLs produced by the AfsA homologs are essential for antibiotic production, and mutation of the corresponding homologous genes lead to impaired or reduced production of the respective antibiotics, while disruption of the cognate receptor genes leads to the opposite or no effect (Takano, 2006; Zou et al., 2014). For example, in *S. hygroscopicus* 5008, inactivation of *afsA* homologs (*shbA1*, *shbA2*, or *shbA3*) dramatically decreased biosynthesis of validamycin; whereas deletion of *arpA* homologs (*shbR1* or *shbR3*) increased validamycin production by 26% (ΔshbR1) and 20% (ΔshbR3), and deletion of *shbR2* had no significant effects on validamycin production (Tan et al., 2015). However, in *S. bingchenggensis*, the SbbR/SbbA system presented an opposite regulatory pattern: inactivation of the GBL synthase gene (*sbbA*) led to an increase of antibiotic production instead of the anticipated reduction or abolishment, while mutation of *sbbR* resulted in reduced milbemycin production. Virtually, titer improvement of milbemycin in *sbbA* mutant strain is not surprising. In *S. bingchenggensis* BC04, SbbR activates *milR* expression by binding to the upstream region of *milR*; expressed *milR* can activate the transcription of biosynthetic structural genes *milA4-E* and *milF*. The putative compound produced by SbbA could directly bind to SbbR, leading to its dissociation from the *milR* promoter. In ΔsbbA, production of the compound might be abolished, and SbbR could bind *milR* promoter persistently and activates its expression, thus leading to an increased milbemycin production. Previously, titer improvements of antibiotic in GBL synthase gene deficiency strains have been reported. In the model strain *S. coelicolor*, deletion of *scbA*, a homolog of *afsA*, led to overproduction of actinorhodin and undecylprodigiosins (Takano et al., 2001; Li et al., 2015). In *S. lividans*, deletion of *scbA* also improved the titers of actinorhodin and undecylprodigiosins (Butler et al., 2003). The mechanisms underlying the role of ScbA in increasing actinorhodin and undecylprodigiosins production in these two strains are unclear, but are supposed to be the molecular events that are closely related with the complex regulatory cascade mediated by GBLs. In this work, inactivation of *sbbA* obviously improved the production of milbemycin by 25% (from 2537 to 3169 mg/l); thus the *sbbA* mutant can be used as a new parental strain in the future genetic engineering to obtain milbemycin high-producing strains.

In this work, SbbR played differential regulatory roles toward its targets: it represses the expression of its own gene and the divergently transcribed *sbbA*, but positively regulates milbemycin biosynthesis by directly activating the transcription of *milR*. The promoter sites covered by SbbR were from nt -106 to -71, nt -30 to +5, and nt -279 to -252 relative to the tsps of *sbbR*, *sbbA*, and *milR*. The auto-repression of SbbR on its own is a common feature exerted by TetR family regulators (Ahn et al., 2012). The mechanism whereby SbbR represses *sbbA* is easy to understand, the protected site overlaps the -35 and -10 elements, SbbR may sterically block the access of RNA polymerase to *sbbA* promoter. While as an activator of *milR*, SbbR locates far upstream of the -35 element and may recruit RNA polymerase to the promoter via direct protein–protein interaction (Rodionov, 2007). GBL receptor homologs that bind (far) upstream of promoter region and act as the direct activator of antibiotic CSRs have been reported previously. For instance, in jadomycin biosynthesis in *S. venezuelae*, the GBL (SVB1) receptor, JadR3, can activate the expression of *jadR1*, the cluster-situated activator of jadomycin biosynthesis. JadR3 has three binding sites in the promoter of *jadR1*. Among these sites, AREII, a region from nt -252 to -219 relative to the *jadR1* tsp, is essential for the activation function of JadR3 (Zou et al., 2014). In *S. ansochromogenes*, SabR binds to the sequences (from nt -64 to -29 relative to the tsp of *sanG*) adjacent to or overlapping partially with the -35 element and positively regulates *sanG* transcription (He et al., 2010). There are also exceptions, AvaR1, the ArpA homolog from *S. avermitilis*, protects two regions (regions from nt -262 to -233 and nt -223 to -193) far upstream of AveR (the cluster-situated activator of avermectins) and acts as a repressor, but not an activator (Zhu et al., 2017); one of the two binding sites (nt -222 to -244 and nt -3 to -35 relative to the *cpkO* tsp) of ScbR from *S. coelicolor* is also far away from *cpkO* promoter and ScbR is a repressor of *cpkO* (Takano et al., 2005). These examples complicate the regulatory mechanisms exerted by GBL receptor homologs. Nevertheless, our study and the observations reported previously lead to the conclusion that, in addition to being a repressor of its own expression and that of other targets, GBL receptor homologs could also interact with the upstream regions of cluster-situated activator genes to promote gene expression, suggesting that a GBL receptor homolog-mediated activation of antibiotic biosynthesis might exist in many streptomycetes.

In the search of SbbR/SbbA regulon, 11 genes that encode seven putative secondary metabolism CSRs and four well-known pleiotropic regulatory homologs have been identified. Of these novel targets, the SARP-family regulators (SBI_08420, SBI_08432, and SBI_09158), the LAL-family regulators (SBI_00827, SBI_01376, and SBI_09325) and Sig24_sbh_ are the potential cluster-situated activators, e.g., SBI_08420, designated as NanR2, has recently been reported to be an essential activator of nanchangmycin production (Yu et al., 2012), suggesting the importance of SbbR/SbbA in the regulation of secondary metabolism. Moreover, homologs of the four pleiotropic regulators GlnR_sbh_, WblA_sbh_, AtrA_sbh_, and MtrA/B_sbh_ have been reported to be involved in the development of *Streptomyces* as well as in the production of various antibiotics in streptomycetes (Rabyk et al., 2011; Lee et al., 2013). Many detailed studies concerning the downstream regulatory networks of these four regulators have been reported (Liu et al., 2013), but little is known about the regulatory proteins that function upstream of these pleiotropic regulators, although AdpA_ch_ from *Streptomyces chattanoogensis* L10 has been confirmed to be a direct activator of *wblA_ch_* (Yu et al., 2014), and AdpA homolog from *Streptomyces roseosporus* directly regulate the *atrA* homolog (Mao et al., 2015). Our finding provides an interesting clue that SbbR, as a GBL receptor homolog, could be a direct controller of these important regulators that act pleiotropically in *Streptomyces*. The molecular regulatory mechanism of SbbR as the upper regulator of these pleiotropic regulators needs further investigation.

SbbR could regulate *milR* directly, however, it is doubted that this is the only regulatory pattern that is exerted by SbbR/SbbA system to regulate milbemycin production. Note that both deletion of *sbbR* and *sbbA* decreased cell biomass in fermentation medium, suggesting the importance of *sbbR* and *sbbA* in cell growth (**Figure 2D**); moreover, SbbR has positive effects on the four well-known pleiotropic regulators (i.e., GlnR_sbh_, WblA_sbh_, AtrA_sbh_, and MtrA/B_sbh_). Therefore, it is possible that SbbR/SbbA system may exert pleiotropic effects on primary and secondary metabolism, and the changes of milbemycin production in ΔsbbR and ΔsbbA is not only due to the changed expression levels of biosynthetic genes, but also the changed supplies of precursors and cofactors. Further studies are required to investigate the regulatory targets of SbbR/SbbA system through comparative transcriptomic analysis among BC04, ΔsbbR, and ΔsbbA, and Chip-seq of SbbR, and thereby gain a more comprehensive overview of the roles of SbbR/SbbA system.

## Author Contributions

HH, YZ, and WX designed the research. HH and YZ wrote the manuscript. HH performed the experiments and LY determined the dry weight and production curves. HW, CL, XG, and XW helped to modify the article. All authors reviewed and approved the manuscript.

## Conflict of Interest Statement

The authors declare that the research was conducted in the absence of any commercial or financial relationships that could be construed as a potential conflict of interest.
