# Supplementary material for: SbbR/SbbA, an Important ArpA/AfsA-Like System, Regulates Milbemycin Production in Streptomyces bingchenggensis
Source: Front Microbiol. 2018 May 23;9:1064. doi: 10.3389/fmicb.2018.01064 (PMC5974925; doi:10.3389/fmicb.2018.01064)
Supplement: Supplementary file 1 [file Data_Sheet_1.docx]

***Supplementary Materials***

SbbR/SbbA, an important ArpA/AfsA-like System, Regulates Milbemycin Production in *Streptomyces bingchenggensis*

Hairong He^1, 2^, Lan Ye^1, 2^, Chuang Li^1, 2^, Haiyan Wang^1^, Xiaowei Guo^2^, Xiangjing Wang^2^, Yanyan Zhang^1^*, Wensheng Xiang^1, 2^*

***Correspondence:**

Yanyan Zhang and Wensheng Xiang

Email: [yyzhang@ippcaas.cn](mailto:yyzhang@ippcaas.cn); [xiangwensheng@neau.edu.cn](mailto:xiangwensheng@neau.edu.cn%20)

**Figures**


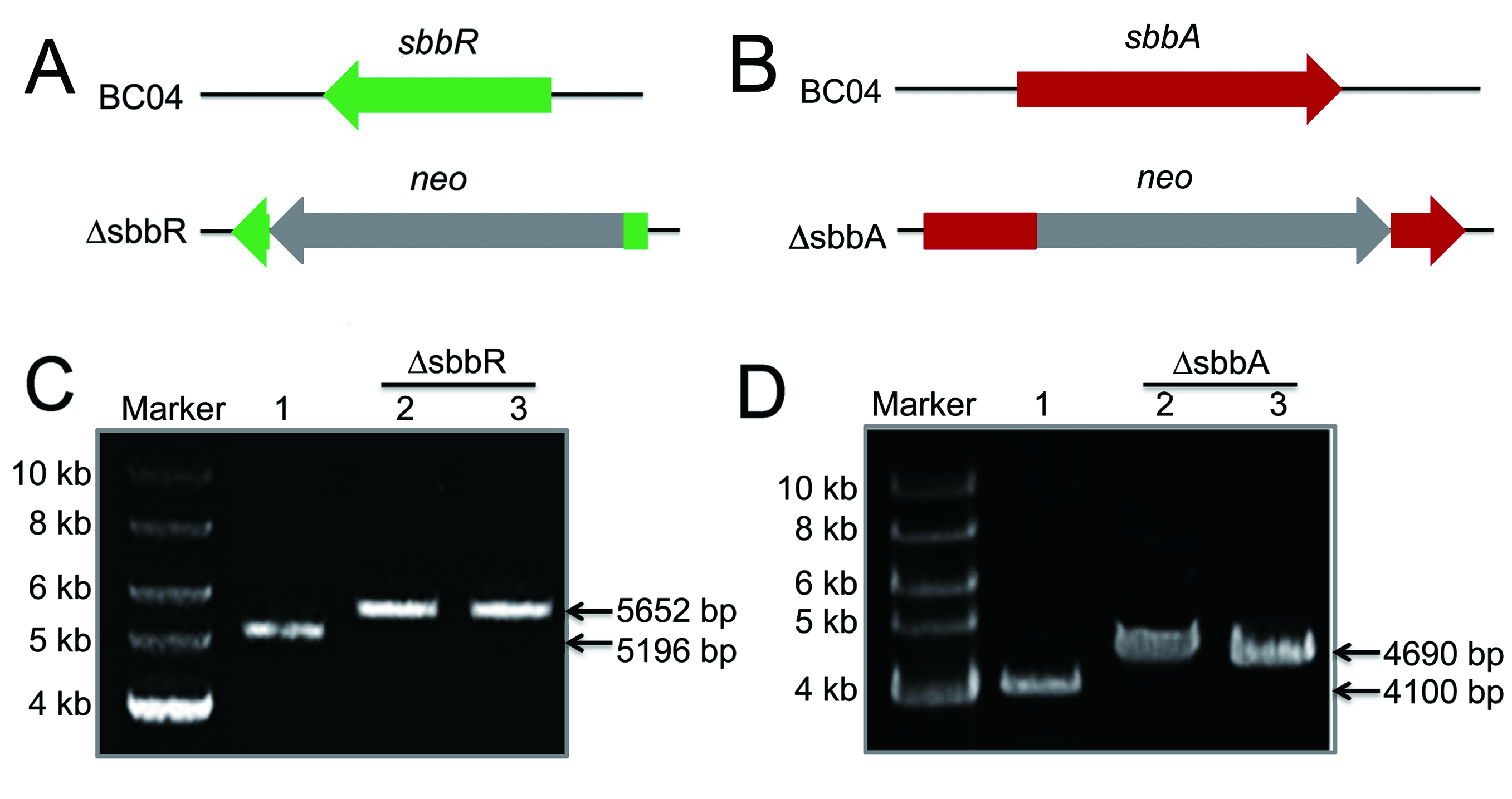


**Fig. S1** **Confirmation of *sbbR* and *sbbA* disruption by PCR amplification.** (A-B) Diagrams of *sbbR* and *sbbA* disruption constructions. (C-D) Agarose gel electrophoresis showing PCR amplified fragments. Line 1 represents the PCR template are the genomic DNA from *S. bingchenggensis* BC04, while lanes 2 and 3 are from two independent mutant clones of ΔsbbR and ΔsbbA, respectively.


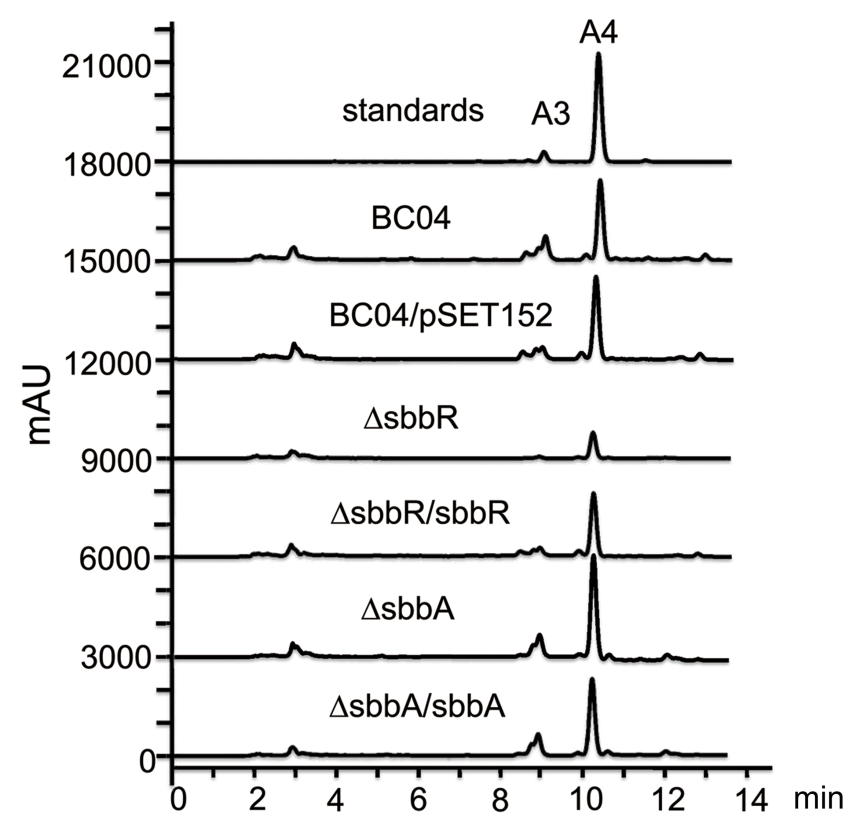


**Fig. S2 HPLC trace of milbemycin A3/A4 production in BC04, BC04/pSET152, ΔsbbR, ΔsbbR/sbbR, ΔsbbA and ΔsbbA/sbbA cultured in fermentation medium for 9 days.**


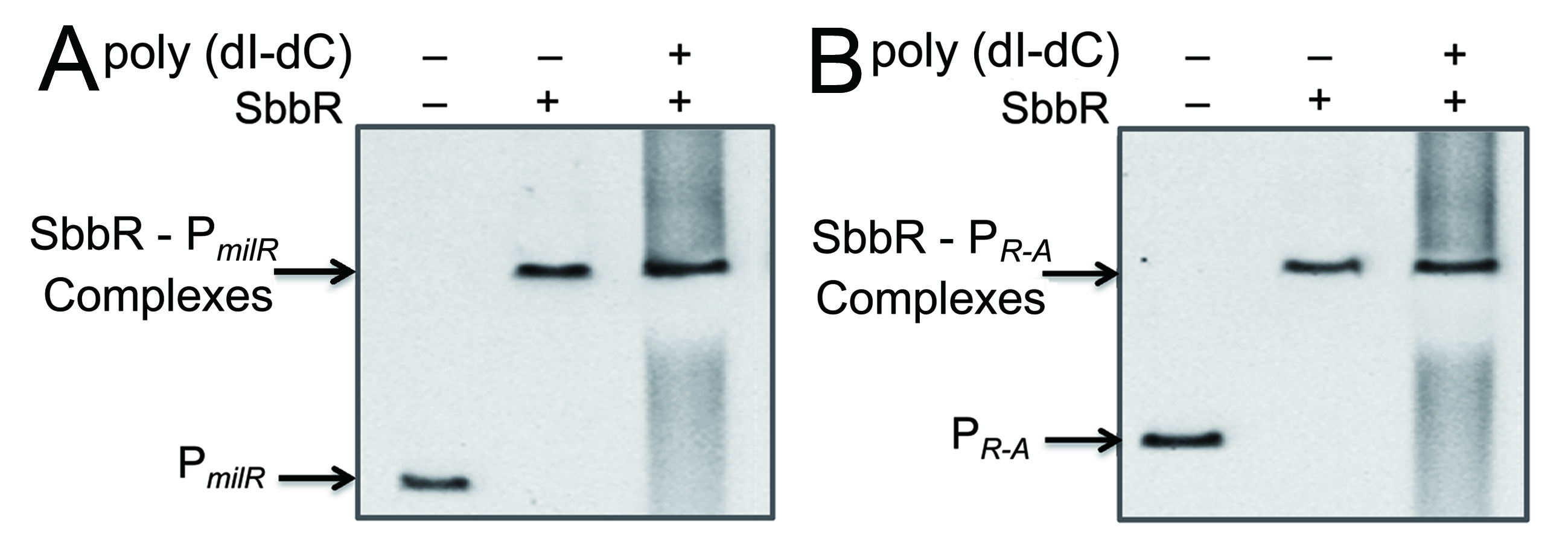


**Fig. S3 Competitive EMSAs for determining the binding of SbbR to the promoter P*_milR_* and P*_R-A_*.** (A) EMSA of SbbR binding to P*_milR_* with 500-fold non-specific poly (dI-dC). Each line contains 10 ng probes. Lane 2-3 contains 0.2 μm SbbR and Line 3 contains 500-fold non-specific poly (dI-dC). DNA-protein complexes and free probes are indicated by arrows. (B) EMSA of SbbR binding to P*_R-A_* with 500-fold non-specific poly (dI-dC).


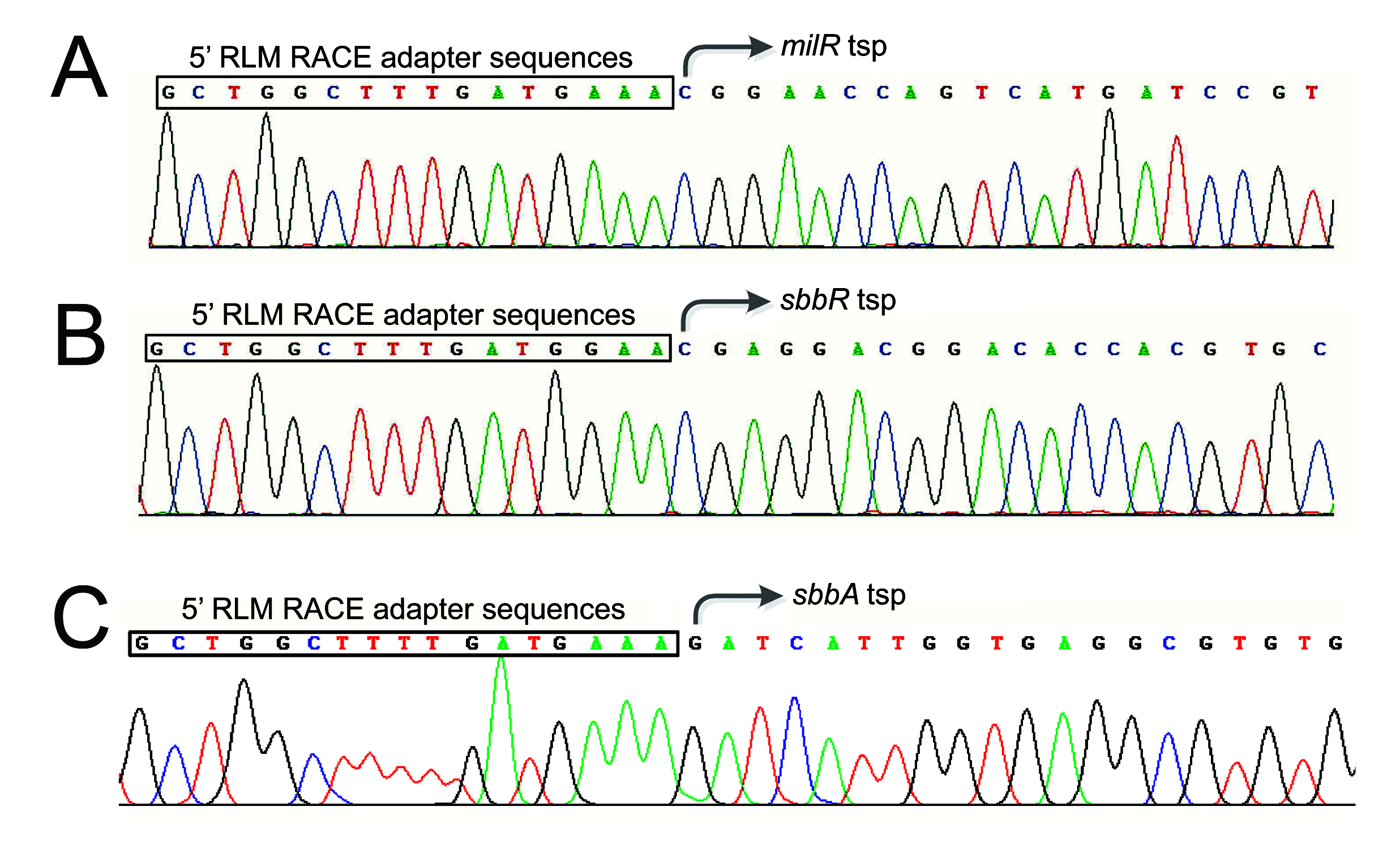


**Fig. S4 Determination of *milR* , *sbbR* and *sbbA* tsp by 5’ RLM-RACE.** (A) 5’ RLM-RACE for *milR*. Sequence of 5’ RACE adaptor is indicated by a box. The tsps are indicated by bent arrows. (B) 5’ RLM-RACE for *sbbR.* (C) 5’ RLM-RACE for *sbbA.*


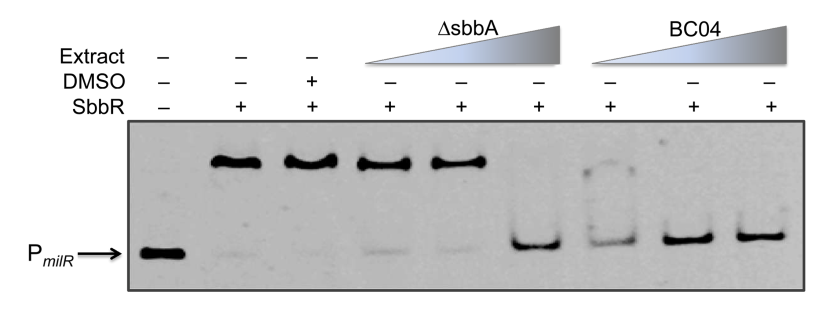


**Fig. S5 Influences of ethyl acetate extracts from ΔsbbA and BC04 on DNA binding activity of SbbR.** EMSAs of SbbR (0.2 μM) on probe P*_milR_* were performed with culture extracts in a series of two-fold dilution steps. DMSO was used as a solvent control.

**Tables**

**Table S1 Primers used in this study.**

| Name | Sequence (5’ to 3’) | | | | | | | | Use |
| --- | --- | --- | --- | --- | --- | --- | --- | --- | --- |
| For gene deletion, complementation, and overexpression | | | | | | |  | |  |
| sbbRLF | CCC*AAGCTT*GGCGTTCGGAGTCCATGAGG, *Hin*dIII | | | | | | | | Deletion of *sbbR* gene |
| sbbRLR | GC*TCTAGA*AGGAGGGTTCGGCGTGTCTG, *Xba*I | | | | | | | |  |
| sbbRRF | CGG*GGTACC*TGCTGCCCTCACTTCAGACCC, *Kpn*I | | | | | | | |  |
| sbbRRR | CG*GAATTC*CGACGGCGAACTGATGTGGC, *Eco*RI | | | | | | | |  |
| CsbbRF | G*GAATTC*CGGGTCGAGCGTGAAGGACA, *Eco*RI | | | | | | | | Complementation of *sbbR* in ΔsbbR |
| CsbbRR | TGC*TCTAGA*AACGACAAGGCGGGCAACG, *Xba*I | | | | | | | |  |
| VsbbRF | GACGAAGATCGCCGATGCCA | | | | | | | | Confirmation of *sbbR* deletion in ΔsbbR |
| VsbbRR | CTGCCCAACAACAACTCCTCCA | | | | | | | |  |
| sbbALF | CCC*AAGCTT*CAACGCCAACGACTGGAACCG, *Hin*dIII | | | | | | | | Deletion of *sbbA* gene |
| sbbALR | GC*TCTAGA*GGGTTTCGGCGACCATCAGG, *Xba*I | | | | | | | |  |
| sbbARF | CGG*GGTACC*TTCTGGTGGTGCGTGAGGCG, *Kpn*I | | | | | | | |  |
| sbbARR | *GATATC*ACCAACGGACAGGGCAAGCAG, *Eco*RV | | | | | | | |  |
| CsbbAF | GC*TCTAGA*CCAGGTGGCGAAGTGTCCCT, *Xba*I | | | | | | | | Complementation of *sbbA* in ΔsbbA |
| CsbbAR | CGTTCGGAGTCCATGAGGGTG, *Eco*RV | | | | | | | |  |
| VsbbAF | CCGTACCCGGCGTTAGTCAT | | | | | | | | Confirmation of *sbbA* deletion in ΔsbbA |
| VsbbAR | AGAAGTCCGGTGCGAAGTCAG | | | | | | | |  |
| prosbbRF | A*CATATG*CAAGAGCGGGCCGAGC, *Nde*I | | | | | | | | Overexpression of His-tagged SbbR in *E.coli* |
| prosbbRR | A*CTCGAG*AGCCTGTCGGGCCATCGG, *Xho*I | | | | | | | |  |
| For EMSAs |  | | | | | | | |  |
| pmilA1-F-2 | GGAGTGGCTGCGTGTACG | | | | | | | | Probe P*_milA1_* |
| pmilA1-R-2 | GGAGATGTCCTTCCCGGTC | | | | | | | |  |
| pmilA2-F-2 | TTCAGGTGGACTTCGACAAGG | | | | | | | | Probe P*_milA2_* |
| pmilA2-R-2 | CGACAATGGCGATGGGTTC | | | | | | | |  |
| pmilA3-F-2 | TCCTCCGGCGATCCCACT | | | | | | | | Probe P*_milA3_* |
| pmilA3-R-2 | GCCAACGGCAACTCGAACC | | | | | | | |  |
| pmilA4-F-2 | CAACACCTTGGCCAGGTCC | | | | | | | | Probe P*_milA4_* |
| pmilA4-R-2 | CGGATTCGGGTCGTACAGC | | | | | | | |  |
| pmilE-F-2 | GCGATGGGCGGCTACTTC | | | | | | | | Probe P*_milE_* |
| pmilE-R-2 | ACCAGATCCGGGTGGGTG | | | | | | | |  |
| Porf1-F | GCTCTAGATTACGGCATCTCCTCGACATTCCTCA | | | | | | | | Probe P*_orf1_* |
| Porf1-R | GGCTACCGCCTCTCGGGAGGAG | | | | | | | |  |
| pmilF-F-2 | GCCACGGTGGTGCTCACG | | | | | | | | Probe P*_milF_* |
| pmilF-R-2 | GCAAGGTCCGCCCTGATG | | | | | | | |  |
| PmilR-F | CCATGTCGATGACACGCCTGAA | | | | | | | | Probe P*_milR_* |
| PmilR-R | GGCCCTCCAGGACTGCTGTCACG | | | | | | | |  |
| PsbbR-A-F | TCGCGTAGTGGAAGTAGATGGC | | | | | | | | Probe P*_R-A_* |
| PsbbR-A-R | CATGCGGGAACGTGGGAT | | | | | | | |  |
| Psbi_00508-F | TGATGTCCCGTGCGTGTCG | | | | | | | | Probe P*_sbi_00508_* |
| Psbi_00508-R | GCGCGGTCGACGACGTACT | | | | | | | |  |
| Psbi_00605-F | GAACGAGGTCACGTCGAGC | | | | | | | | Probe P*_sbi_00605_* |
| Psbi_00605-R | CGCTTTGCGGGACTCATC | | | | | | | |  |
| Psbi_01006-F | CGCCAGGACGGAGACGAA | | | | | | | | Probe P*_sbi_01006_* |
| Psbi_01006-R | GACACGCTGTGCGGAGGG | | | | | | | |  |
| Psbi_01043-F | CCGCACCCCGAGGAACACCT | | | | | | | | Probe P*_sbi_01043_* |
| Psbi_01043-R | ATGCTCGCCCCACAGCACC | | | | | | | |  |
| Psbi_01144-F | AGCCGCGAGGAAGTATCGC | | | | | | | | Probe P*_sbi_01144_* |
| Psbi_01144-R | GTGGGAGCACTGCGTTGACA | | | | | | | |  |
| Psbi_01367-F | ATGCCTGGTCGAATGACGTCT | | | | | | | | Probe P*_sbi_01367_* |
| Psbi_01367-R | CGTCTTCGACTACCTGGACGG | | | | | | | |  |
| Psbi_03002-F | TCGGGGTCAGTCGGCTCG | | | | | | | | Probe P*_sbi_03002_* |
| Psbi_03002-R | GCACCGTCCGGCTGTGGA | | | | | | | |  |
| Psbi_03913-F | GGCGAACTTGACCAGTTCATA | | | | | | | | Probe P*_sbi_03913_* |
| Psbi_03913-R | GGCCTCGTCGGCTATGAC | | | | | | | |  |
| Psbi_06432-F | GTGACCACCTCGCCCTCGG | | | | | | | | Probe P*_sbi_06432_* |
| Psbi_06432-R | GTCGGTCATCGCCGCCAG | | | | | | | |  |
| Psbi_06842-F | GAGCATCTGGTTGGCGTACA | | | | | | | | Probe P*_sbi_06832_* |
| Psbi_06842-R | GGTCCTCGATGACCAAGTCG | | | | | | | |  |
| Psbi_06991-F | GGCCTGCCGACTCATTTC | | | | | | | | Probe P*_sbi_06991_* |
| Psbi_06991-R | CAGAAGGGCGGTGAGAATG | | | | | | | |  |
| PnanR1R2F | GGCAGGACGGCGTTCTCG | | | | | | | | Probe P*_nanR1R2_* |
| PnanR1R2R | TCTTCGCTGCTCACCACCTCG | | | | | | | |  |
| Psbi_08432-F | ACCAGCAGGCTCAGCAACCG | | | | | | | | Probe P*_sbi_08432_* |
| Psbi_08432-R | ACGCCACAGCCACGACGAA | | | | | | | |  |
| Psbi_08948-F | CCAGCAGTGCACGCAGCC | | | | | | | | Probe P*_sbi_08948_* |
| Psbi_08948-R | GAGCGTATGCACGGCCAGAC | | | | | | | |  |
| Psbi_09158-F | CGGAACGAGCGAGCAGCA | | | | | | | | Probe P*_sbi_09158_* |
| Psbi_09158-R | GCGCCAGGCGAGATGGAC | | | | | | | |  |
| Psbi_00827-F | GCAACCGCCGGTACAGCT | | | | | | | | Probe P*_sbi_00827_* |
| Psbi_00827-R | CGAAACAGGCGTGCCAGC | | | | | | | |  |
| Psbi_01034-F | CAGATAGGTCGCCGAGAAGG | | | | | | | | Probe P*_sbi_01034_* |
| Psbi_01034-R | AGCCGTGTCTTGCCGATG | | | | | | | |  |
| Psbi_01376-F | GGTCCCGGACCACTTCGT | | | | | | | | Probe P*_sbi_01376_* |
| Psbi_01376-R | GGTTGTAGTGCCGCTCGTAG | | | | | | | |  |
| Psbi_02794-F | GGACGACGGCAGCACCCT | | | | | | | | Probe P*_sbi_02794_* |
| Psbi_02794-R | CCTGCATCAGCCGGGTCT | | | | | | | |  |
| Psbi_03563-F | ATCCGCCGCCGATGAACC | | | | | | | | Probe P*_sbi_03563_* |
| Psbi_03563-R | TCCCCTCCGACGAGCAGC | | | | | | | |  |
| Psbi_05042-F | GTCCACGAGCACCAGCAGC | | | | | | | | Probe P*_sbi_05042_* |
| Psbi_05042-R | TCGAGCAGGGCGGTCTTG | | | | | | | |  |
| Psbi_09026-F | CTCGCCGGTCTTCTCCACC | | | | | | | | Probe P*_sbi_09026_* |
| Psbi_09026-R | CTCCAGCGCCTCGCTCAC | | | | | | | |  |
| Psbi_09029-F | TTCGAACTGCACGTCCGC | | | | | | | | Probe P*_sbi_09029_* |
| Psbi_09029-R | AAGTCGTCTTGCCGATACCC | | | | | | | |  |
| Psbi_09031-F | CGCCGTACCGCTGAGGGTGA | | | | | | | | Probe P*_sbi_09031_* |
| Psbi_09031-R | ACCGTGAGATAGCGGAGTCGC | | | | | | | |  |
| Psbi_09325-F | CACCAGCATGAGCAGAACCC | | | | | | | | Probe P*_sbi_09325_* |
| Psbi_09325-R | GCAGAGCGGCGATGACGG | | | | | | | |  |
| Psbi_02074-F | TTCGGCCTTCGCAGCCGT | | | | | | | | Probe P*_sbi_02074_* |
| Psbi_02074-R | GTCAGCACCTTTTCACTCACCC | | | | | | | |  |
| Psbi_02069-F | CATGCCAGTACACGGTGTTGA | | | | | | | | Probe P*_sbi_02069_* |
| Psbi_02069-R | GCTATGCGGAGTCCGAGCT | | | | | | | |  |
| Psbi_02067-F | GCCCAGCGACCACAATCT | | | | | | | | Probe P*_sbi_02067_* |
| Psbi_02067-R | GCATCTGCGGAACGGACTT | | | | | | | |  |
| Psbi_02061-F | GATCGCGGCGGTCAGCGG | | | | | | | | Probe P*_sbi_02061_* |
| Psbi_02061-R | GCCCAGGACGCGGGCGTA | | | | | | | |  |
| Psbi_02065-F | CTCCACCGGAGCCCATGTCA | | | | | | | | Probe P*_sbi_02065_* |
| Psbi_02065-R | GGCCAGCGTCCGCTTGAGTA | | | | | | | |  |
| Psbi_02592-F | TCGCCTGGCTGACATCGC | | | | | | | | Probe P*_sbi_02592_* |
| Psbi_02592-R | CCTGACGCCCTCCACTTCG | | | | | | | |  |
| Psbi_02748-F | GGCGAGGGGTCTGAGCCT | | | | | | | | Probe P*_sbi_02748_* |
| Psbi_02748-R | GGTGGGTGGGGAAGTAGCG | | | | | | | |  |
| Psbi_03920-F | TGCCGTCCTCGTGGGTGA | | | | | | | | Probe P*_sbi_03920_* |
| Psbi_03920-R | GCCGGTGAGCAATCGGAGC | | | | | | | |  |
| Psbi_08135-F | GGCTCCTCCCAGCGGTAG | | | | | | | | Probe P*_sbi_08135_* |
| Psbi_08135-R | CGGTCAGTGCCTGCTTCTCA | | | | | | | |  |
| Psbi_09644-F | ACGAGCGGCCAAAGGGAT | | | | | | | | Probe P*_sbi_09644_* |
| Psbi_09644-R | CTCGATGAGCACGTTGAGGAT | | | | | | | |  |
| Psbi_09685-F | GGCTTGAGTGCCCGTTCG | | | | | | | | Probe P*_sbi_09685_* |
| Psbi_09685-R | ACGGCTGCCAGGTCATCG | | | | | | | |  |
| Psbi_09665-F | GTGCTCGACCAACTCGTCCC | | | | | | | | Probe P*_sbi_09665_* |
| Psbi_09665-R | CGAACCTCGTCCGGCTCA | | | | | | | |  |
| Psbi_06451-F | GTACATCCAGGACGCGATCC | | | | | | | | Probe P*_sbi_06451_* |
| Psbi_06451-R | ATTGACCAGCGCCTTTCG | | | | | | | |  |
| Psbi_06463-F | CGCCGCTCCCAGAAGGAT | | | | | | | | Probe P*_sbi_06463_* |
| Psbi_06463-R | CACGCCGAGGTCGCTCAT | | | | | | | |  |
| Psbi_06838-F | GGCGATCATTCGGCAGGAA | | | | | | | | Probe P*_sbi_06838_* |
| Psbi_06838-R | TCGAGGACCGCGACATCC | | | | | | | |  |
| Psbi_06885-F | ATCGGCGGTGTCGGCAAGGT | | | | | | | | Probe P*_sbi_06885_* |
| Psbi_06885-R | TACCAGCGGGCGTAGGTCTCCT | | | | | | | |  |
| Psbi_05975-F | CAAGCTGCTCGCGGTGTT | | | | | | | | Probe P*_sbi_05975_* |
| Psbi_05975-R | TGTCGGCTATCCCGTCCC | | | | | | | |  |
| Psbi_06839-F | GTGGCGAAGGTGCTGGTG | | | | | | | | Probe P*_sbi_06839_* |
| Psbi_06839-R | CGCGACGCAGAAGTAGCC | | | | | | | |  |
| Psbi_05051-F | TCCAGAGCACCCGTGAAACC | | | | | | | | Probe P*_sbi_05051_* |
| Psbi_05051-R | GCGCACCTGGGGCAGATC | | | | | | | |  |
| Psbi_03966-F | TGTTCCCGGTGCTGTTGC | | | | | | | | Probe P*_sbi_03966_* |
| Psbi_03966-R | TCTGCTGAATGCCGTTCCA | | | | | | | |  |
| Psbi_02912-F | GCCATGGACTCCTGTGCG | | | | | | | | Probe P*_sbi_02912_* |
| Psbi_02912-R | CCTTGTCGACCAGCGACTG | | | | | | | |  |
| Psbi_08337-F | TCGAGTACTTCGCCGAGACG | | | | | | | | Probe P*_sbi_08337_* |
| Psbi_08337-R | GGACGAGTCGCCGCTGAC | | | | | | | |  |
| Psbi_08362-F | GGTGCTGAGGTCCTCGTAGCC | | | | | | | | Probe P*_sbi_08362_* |
| Psbi_08362-R | AGCCAGGAGCGGCGGTTA | | | | | | | |  |
| Psbi_0241-F | AGGTGCCGATCAGGACGAA | | | | | | | | Probe P*_sbi_00241_* |
| Psbi_0241-R | GCGTGGAGATCAGCCCTTG | | | | | | | |  |
| Psbi_08510-F | CGAAAGTTCCGGGCGATC | | | | | | | | Probe P*_sbi_08510_* |
| Psbi_08510-R | GCAACGGGTTGAGCAGGC | | | | | | | |  |
| Psbi_05821-F | CTTCAGCGGCTTTAGCTGTTT | | | | | | | | Probe P*_sbi_05821_* |
| Psbi_05821-R | GTGCGGTGGAGCTTCACTTT | | | | | | | |  |
| Psbi_06304-F | ACTTCGTGGTAGCGAATTGTGA | | | | | | | | Probe P*_sbi_06304_* |
| Psbi_06304-R | GTGAAGCGGACACGGAACA | | | | | | | |  |
| Psbi_08091-F | GGTCGCCCCTGTTCGTCG | | | | | | | | Probe P*_sbi_08091_* |
| Psbi_08091-R | CGCCCGCACGACCTTCAC | | | | | | | |  |
| Psbi_06321-F | TACGCCATCTGCGAACGG | | | | | | | | Probe P*_sbi_06321_* |
| Psbi_06321-R | CAGGGCATCGACCAACTCC | | | | | | | |  |
| Psbi_02246-F | CCTGGGGTTCATCCTCTTCAGC | | | | | | | | Probe P*_sbi_02246_* |
| Psbi_02246-R | GGTCCGCCGTGCTGTGCT | | | | | | | |  |
| Psbi_05610-F | CCGGTCGACGAGTTGATC | | | | | | | | Probe P*_sbi_05610_* |
| Psbi_05610-R | AGGAACGGGCGTATAGCG | | | | | | | |  |
| Psbi_04933/4-F | GCACCCTCCTCGCACGCA | | | | | | | | Probe P*_sbi_04933_* |
| Psbi_04933/4-R | CGCCTTGGCCAGCATCGT | | | | | | | |  |
| Psbi_06494/5-F | GGATTCGGGTCGTTCTCAAGC | | | | | | | | Probe P*_sbi_06494_* |
| Psbi_06494/5-R | GCGAACGGATCGCCTCCA | | | | | | | |  |
| Psbi_04522-F | CGGCGATCAGATACAGCACG | | | | | | | | Probe P*_sbi_04522_* |
| Psbi_04522-R | AGCACCAGGACGGGGAAGC | | | | | | | |  |
| Psbi_04643-F | GCCGCCTTGTCACTGATCC | | | | | | | | Probe P*_sbi_04643_* |
| Psbi_04643-R | ACGTCGATGTCATCGCTCC | | | | | | | |  |
| Psbi_02366-F | GATGCCGAGGAGCACCAC | | | | | | | | Probe P*_sbi_02366_* |
| Psbi_02366-R | CGGGACGCCGTAGAAGTC | | | | | | | |  |
| Psbi_02354-F | AAGGGCTGCGACCTGCTG | | | | | | | | Probe P*_sbi_02354_* |
| Psbi_02354-R | CGCCTTGGTCGTCACCTCA | | | | | | | |  |
| Psbi_06792-F | AGGGCTCGGTCCAGAACA | | | | | | | | Probe P*_sbi_06792_* |
| Psbi_06792-R | GCGGACCAGGGACACGAT | | | | | | | |  |
| Psbi_04010-F | ACGGAGCTGGTCGGGATT | | | | | | | | Probe P*_sbi_04010_* |
| Psbi_04010-R | GCGGCGGAGTACATCTGGT | | | | | | | |  |
| Psbi_03112-F | ATGCGACGGCGGACGTGG | | | | | | | | Probe P*_sbi_03112_* |
| Psbi_03112-R | TCGGGGTGACCTCGACCGT | | | | | | | |  |
| Psbi_0I9715-F | TGGACCACCGAGACGCTG | | | | | | | | Probe P*_sbi_09715_* |
| Psbi_09715-R | GACGGACCGCCTGGACTC | | | | | | | |  |
| Psbi_03475-F | CATGAACATTCGTTTCTGCTGC | | | | | | | | Probe P*_sbi_03475_* |
| Psbi_03475-R | CCAACTGGCGTGCTCTGC | | | | | | | |  |
| Psbi_01075-F | TCCAGCAGGCACTTGGCG | | | | | | | | Probe P*_sbi_01075_* |
| Psbi_01075-R | CCTCGATGGTCACGACTCGAC | | | | | | | |  |
| Psbi_02438-F | CCGTTTCGGCGTAGTCCT | | | | | | | | Probe P*_sbi_02438_* |
| Psbi_02438-R | ATCCAAAATGGTCCGAGAAC | | | | | | | |  |
| Psbi_05779-F | TGGCGTGGCGAGATCGATC | | | | | | | | Probe P*_sbi_05779_* |
| Psbi_05779-R | CCTCGGCTATACGACGGACCAG | | | | | | | |  |
| Psbi_05811-F | TCCCGCGAGCACGCTGAC | | | | | | | | Probe P*_sbi_05811_* |
| Psbi_05811-R | CCGCTCACGCTCGGTCATG | | | | | | | |  |
| Psbi_06911F | GACATGGGCGCACCGGAATC | | | | | | | | Probe P*_sbi_06911_* |
| Psbi_06911R | ATAGGGCCGCCGCTGAAGAG | | | | | | | |  |
| For DNase I footprinting | | | | | |  | |  | |
| FAM-pmilR-F | CCATGTCGATGACACGCCTGAA | | | | | | | | *milR* promoter region |
| HEX-pmilR-R | GGCCCTCCAGGACTGCTGTCACG | | | | | | | |  |
| FAM-psbbA-F | TCGCGTAGTGGAAGTAGATGGC | | | | | | | | *sbbR* promoter region |
| HEX-psbbA-R | CATGCGGGAACGTGGGAT | | | | | | | |  |
| For mutation probes P*_milR_* and P*_R-A_* | | | |  | | | | |  |
| mupmilR-F | GAATTCACAATCATACTAGTCATTTCTTGTCTCCCCTTGT | | | | | | | | Converse site mutation in P*_milR_* |
| mupmilR-R | ACATAGGGACGGTCTCGACCA | | | | | | | |  |
| mupsbbA-F | GAATTCTTACTAGTATTACTAGTCGATCATTGGTGAGGCGTGTGG | | | | | | | | Converse site mutation in P*_R-A_* |
| mupsbbA-R | ACCTGTGGGTCTTCACCTCCA | | | | | | | |  |
| For qRT-PCR |  | | | | | | | |  |
| real-16sF | TGTCGTGAGATGTTGGGTTAAG | | | | | | | | *16s* ORF |
| real-16sR | TCATTGTACCGGCCATTGTAG | | | | | | | |  |
| real-milRF | GCAAGAGCGAATTCCTGAAG | | | | | | | | *milR* ORF |
| real-milRR | CTGTGACGGAGCGAGTC | | | | | | | |  |
| real-sbbRF | GCACCAGCATCAGCGATA | | | | | | | | *sbbR* ORF |
| real-sbbRR | AGCGCGAGTTTCTCCTTG | | | | | | | |  |
| real-sbbAF | CCTGTGGGTCCAGGAAGCG | | | | | | | | *sbbA* ORF |
| real-sbbAR | AACACGGGGCCACGGATG | | | | | | | |  |
| real-milA3F | CGCGTATATGTCAGAGGATCAG | | | | | | | | *milA3* ORF |
| real-milA3R | AAGTGGAATGGTGTGCAGT | | | | | | | |  |
| real-milA4F | TTGAGGGCTATGCGATGAC | | | | | | | | *milA4* ORF |
| real-milA4R | ACGACTGACACGCCAAAT | | | | | | | |  |
| real-milFF | CGCTGAACATCCTGGTGAA | | | | | | | | *milF* ORF |
| real-milFR | CCGTACATTGACGCTGAAGA | | | | | | | |  |
| real-8420F | GAGGTGGTGAGCAGCGAAGA | | | | | | | | *sbi_08420* ORF |
| real-8420R | TTGCGGAGTTGGGACACG | | | | | | | |  |
| real-8432F | GGAGAACCACTGCGTGAAA | | | | | | | | *sbi_08432* ORF |
| real-8432R | TTCCCTGAAATGCCGGATG | | | | | | | |  |
| real-9158F | GTTGACGGACCGCAATCT | | | | | | | | *sbi_09158* ORF |
| real-9158R | GATACGCGGAGTTGGGAAG | | | | | | | |  |
| real-827F | CCTTCGACGAGGCATTCG | | | | | | | | *sbi_00827* ORF |
| real-827R | CATCGCCCTTGTTCCACATA | | | | | | | |  |
| real-1376F | CGGATGGGCAACTATCAGG | | | | | | | | *sbi_01376* ORF |
| real-1376R | TTGATGGCCAGGTTGTAGTG | | | | | | | |  |
| real-9325F | GCTGTGGCAGGACTTCAA | | | | | | | | *sbi_09325* ORF |
| real-9325R | AGCGCATAGACATGGTTGTT | | | | | | | |  |
| real-6451F | AAGGTGTGGCCGAAGTG | | | | | | | | *sbi_06451* ORF |
| real-6451R | ACGAGGCATGCGTATTGA | | | | | | | |  |
| real-5051F | GAACGTGGGCTACCGATTC | | | | | | | | *sbi_05051* ORF |
| real-5051R | ATGTTCTCCGCCATGTTCTC | | | | | | | |  |
| real-5811F | TCCCACGGTCACCTCTT | | | | | | | | *sbi_05811* ORF |
| real-5811R | GGCGAAGCGCTCGTATT | | | | | | | |  |
| real-5779F | ACTCCACCTCGACCCCGACC | | | | | | | | *sbi_05779* ORF |
| real-5779R | CGTATAGCCGAGGAGGAGACCG | | | | | | | |  |
| real-6494F | CTACATCGTCAAGCCGTTCA | | | | | | | | *sbi_06494* ORF |
| real-6494R | CACATCGATCACCAGATCCC | | | | | | | |  |
| For GFP reporter system | | |  | |  | | | | |
| pmilRGFP-F | CG*GGATCC*CCATGTCGATGACACGCCTGAA, *Bam*HI | | | | | | | | P*_milR_* |
| pmilRGFP-R | GGCCCTCCAGGACTGCTGTCACG | | | | | | | |  |
| psbbAGFP-F | CG*GGATCC*CGCGTAGTGGAAGTAGATGGC, *Bam*HI | | | | | | | | P*_sbbA_* |
| psbbAGFP-R | GAGCTCCCACGGCTCCGGGAC | | | | | | | |  |
| GFP-F | GATTTCTGGAAACTAGAAGGAGG | | | | | | | | Green fluorescence gene (*gfp*) |
| GFP-R | GC*TCTAGA*AGAGAGTCACTAAGGGCTAACTAAC, *Xba*I | | | | | | | |  |
| PSF14-F | **GATCCCCGGGGACCTGCAGGTCGACTCTAGCTA**GGCCTTGACCTTGATGAGGCG | | | | | | | | Assembly of SF14-driven *sbbR* |
| PSF14-R | **ATGGGTCCTCCTGTGGAGTGGTTCTGTGGATCCCTAATCGAGTATTGATTGTAG** | | | | | | | |  |
| psbbRGFP-F | **TGAGCTACAATCAATACTCGATTAGGGATCC**CATATGCAAGAGCGGGCCGAGC | | | | | | | |  |
| psbbRGFP-R | **TGAACTCACCGCGACGTATCGGGCCCTGGCCAG**TCAAGCCTGTCGGGCCATCGG | | | | | | | |  |
| For 5’ RLM RACE | |  | | | | | | |  |
| 5’ RLM RACE outer | GCTGATGGCGATGAATGAACACTG | | | | | | | | Determination of the tsp of *milR* |
| 5’ race milR outer | TCCGTGCCGATCAGCAGGAT | | | | | | | |  |
| 5’ RLM RACE inner | CGC*GGATCC*GAACACTGCGTTTGCTGGCTTTGATG, *Bam*HI | | | | | | | |  |
| 5’ race milR inner | CGG*GGTACC*AGCGAGCGTGGTGGGCGATA, *Kpn*I | | | | | | | |  |
| 5’ race sbbR outer | TCGCGTAGTGGAAGTAGATGGC | | | | | | | | Determination of the tsp of *sbbR* |
| 5’ race sbbR inner | GGCGTAGCCCCGTTCGTC | | | | | | | |  |
| 5’ race sbbA outer | GGTCGCTCCAGTGGTCGAAGAA | | | | | | | | Determination of the tsp of *sbbA* |
| 5’ race sbbA inner | CGCCTCACGCACCACCAGAA | | | | | | | |  |

**Table S2 Promoters of these genes were used in EMSAs with SbbR, the underlined ones are putative targets for SbbR.**

| # | Locus tag | Gene | Function |
| --- | --- | --- | --- |
| Genes in the milbemycins biosynthetic gene cluster | | | |
| 01 | sbi_00726 | *milA2* | Polyketide synthase modules |
| 02 | sbi_00728 | *milE* | Cytochrome P450 |
| 03 | sbi_00729 | *milA4* | Polyketide synthase modules |
| 04 | sbi_00730 | *orf1* | Dehydrogenases with different specificities (related to short-chain alcohol dehydrogenases |
| 05 | sbi_00731 | *milF* | C5-ketoreductase |
| 06 | sbi_00733 | *milA3* | Polyketide synthase modules |
| 07 | sbi_00734 | *milR* | LAL family regulator |
| 08 | sbi_00789 | *milA1* | Type I polyketide synthase modules |
| SARP family transcriptional regulators | | | |
| 09 | sbi_00508 |  | DNA-binding transcriptional activator of SARP family (cyclase) |
| 10 | sbi_00605 |  | Isocitrate/isoproplmalate dehydrogenase (peptidase) |
| 11 | sbi_01006 |  | DNA-binding transcriptional activator of SARP family |
| 12 | sbi_01043 |  | DNA-binding transcriptional activator of SARP family, AfsR family regulator |
| 13 | sbi_01144 |  | Tetratricopeptide TPR_2 repeat protein |
| 14 | sbi_01367 |  | Predicated ATPase, transcriptional regulator |
| 15 | sbi_03002 |  | Predicated ATPase, XRE family transcriptional regulator |
| 16 | sbi_03913 |  | Response regulators consisting of a CheY-like receiver domain and a winged-helix DNA binding domain, hypothetical protein |
| 17 | sbi_06432 |  | Transcriptional regulators containing DNA-binding HTH domain and aminotransferase domain, GntR family transcriptional regulator |
| 18 | sbi_06842 |  | DNA-binding transcriptional activator of SARP family, hypothetical protein |
| 19 | sbi_06991 |  | Response regulators consisting of a CheY-like receiver domain and a winged-helix DNA binding domain |
| 20 | sbi_08420 | *nanR2* | DNA-binding transcriptional activator of the SARP family |
| 21 | sbi_08432 |  | Response regulator containing CheY-like receiver and SARP domains |
| 22 | sbi_08948 |  | Predicated ATPase, AfsR family regulator |
| 23 | sbi_09158 |  | DNA-binding transcriptional activator of SARP family |
| LAL family transcriptional regulators | | | |
| 24 | sbi_00827 |  | DNA-binding HTH domain-containing protein, LuxR family transcriptional regulator |
| 25 | sbi_01034 |  | Predicated ATPase, transcriptional regulator winged helix family protein |
| 26 | sbi_01376 |  | DNA-binding HTH domain-containing protein, LuxR family transcriptional regulator |
| 27 | sbi_02794 |  | Predicated ATPase, Helix turn helix transcriptional regulator |
| 28 | sbi_03563 |  | Predicated ATPase, LuxR family transcriptional regulator |
| 29 | sbi_05042 |  | Predicated ATPase, LuxR family transcriptional regulator |
| 30 | sbi_09026 |  | Predicated ATPase, protein kinase, LuxR family transcriptional regulator |
| 31 | sbi_09029 |  | Predicated ATPase, LuxR family transcriptional regulator |
| 32 | sbi_09031 |  | Predicated ATPase, LuxR family transcriptional regulator |
| 33 | sbi_09325 |  | Predicated ATPase, LuxR family transcriptional regulator |
| Regulatory gene in the secondary metabolism cluster | | | |
| 34 | sbi_02074 |  | Predicted transcriptional regulator, HxlR family regulator |
| 35 | sbi_02069 |  | Transcriptional regulator, hypothetical protein M271_06645 |
| 36 | sbi_02067 |  | cAMP-binding protein-catabolite gene activator and regulatory subunit of cAMP-dependent protein kinase, Crp/Fnr family transcriptional regulator |
| 37 | sbi_02061 |  | AsnC family transcriptional regulator |
| 38 | sbi_02065 |  | Serine/threoine protein kinase |
| 39 | sbi_02592/3 |  | Response regulators consisting of a CheY-like receiver domain and a winged-helix DNA binding domain |
| 40 | sbi_02747/8 |  | TetR family transcriptional regulator |
| 41 | sbi_03920 |  | PadR family transcriptional regulator |
| 42 | sbi_08135 | *sig50* | SigE family polymerase sigma factor |
| 43 | sbi_09644 |  | IclR family transcriptional regulator |
| 44 | sbi_09685 |  | MarR family transcriptional regulator |
| 45 | sbi_09665 |  | TetR family transcriptional regulator |
| 46 | sbi_06451 | *sigE* | SigE family polymerase sigma factor |
| 47 | sbi_06463 |  | TetR family transcriptional regulator |
| 48 | sbi_06838 |  | Response regulator containing a CheY-like receiver domain and an HTH DNA binding domain, putative two-component system response regulator |
| 49 | sbi_06885 | *sig43* | SigE family polymerase sigma factor |
| 50 | sbi_05975 |  | Response regulators consisting of a CheY-like receiver domain and a winged-helix DNA binding domain |
| 51 | sbi_06839 |  | Two component sensor histidine kinase |
| Homologous protein in streptomycetes | | | |
| 52 | sbi_05051 | *glnR* | Response regulators consisting of a CheY-like receiver domain and a winged-helix DNA binding domain, transcriptional regulator |
| 53 | sbi_03966 | *dasR* | GntR family transcriptional regulator |
| 54 | sbi_02912 | *dasR* | GntR family transcriptional regulator |
| 55 | sbi_08337 | *dasR* | Hypothetical protein |
| 56 | sbi_08362 | *dasR* | GntR family transcriptional regulator |
| 57 | sbi_00241 | *dasR* | GntR family transcriptional regulator |
| 58 | sbi_08510 | *crp* | cAMP-binding protein–catabolite gene activator and regulatory subunit of cAMP-dependent protein kinase, Crp/Fnr family transcriptional regulator |
| 59 | sbi_05821 | *crp* | cAMP-binding protein–catabolite gene activator and regulatory subunit of cAMP-dependent protein kinase, Crp/Fnr family transcriptional regulator |
| 60 | sbi_06304 | *afsK* | Serine/threoine protein kinase |
| 61 | sbi_08091 | *afsK* | Serine/threoine protein kinase |
| 62 | sbi_06321 | *afsR* | Predicated ATPase, AfsR/SARP family transcriptional regulator |
| 63 | sbi_02246 | *afsR* | SAM-dependent methyltransferase |
| 64 | sbi_05610 | *afsR* | Response regulator containing CheY-like receiver and SARP domains, SARP family transcriptional regulator |
| 65 | sbi_04993/4 | *phoP/R* | two-component system, PhoP/R homolog |
| 66 | sbi_06494/5 | *mtrA/B* | two-component system, MtrA/B homolog |
| 67 | sbi_04522 | two-component system | Response regulators consisting of a CheY-like receiver domain and a winged-helix DNA binding domain |
| 68 | sbi_04643 | two-component system | Response regulators consisting of a CheY-like receiver domain and a winged-helix DNA binding domain, two-component system response regulator |
| 69 | sbi_02366 | *bldD* | Predicted XRE transcriptional regulator |
| 70 | sbi_02354 | *metK* | S-adenosylmethionine synthetase |
| 71 | sbi_06792 | *metK* | S-adenosylmethionine synthetase |
| 72 | sbi_04010 | *sigR* | SigE family polymerase sigma factor |
| 73 | sbi_03112 | *rok7B7* | ROK family transcriptional regulator/sugar kinase |
| 74 | sbi_09715 | *rok7B7* | ROK domain containing protein |
| 75 | sbi_03475 | *nsdA* | Hypothetical protein, NsdA |
| 76 | sbi_01075 | *nsdA* | Hypothetical protein, NsdA |
| 77 | sbi_02438 | *nsdA* | Hypothetical protein, NsdA |
| 78 | sbi_05779 | *atrA* | TetR family transcriptional regulator |
| 79 | sbi_05811 | *wblA* | [WhiB family transcriptional regulator](https://blast.ncbi.nlm.nih.gov/Blast.cgi#alnHdr_759777852) |
| 80 | sbi_06911 | *adpA* | AraC family transcriptional regulator |
